# The transcriptional repressor Ctbp2 as a metabolite sensor regulating cardiomyocytes proliferation and heart regeneration

**DOI:** 10.1186/s10020-025-01168-8

**Published:** 2025-03-26

**Authors:** Yanting Meng, Jianwen Ding, Yanping Wang, Jing Wang, Wei Huang, Wenkang Jiang, Jiayi Li, Xiujuan Lang, Sifan Zhang, Yumei Liu, Xijun Liu, Hulun Li, Bo Sun

**Affiliations:** 1 Department of Neurobiology, School of Basic Medical Sciences, Harbin Medical University, 157 Health Road, Nangang District, Harbin, 150081 Heilongjiang China; 2 The Key Laboratory of Myocardial Ischemia, Harbin Medical University, Ministry of Education, 157 Health Road, Nangang District, Harbin, 150081 Heilongjiang China

**Keywords:** Ctbp2, Myocardial infarction, Cardiomyocyte proliferation, NADH/NAD+, Fatty acyl-CoA

## Abstract

**Background:**

C-terminal binding protein-2 (Ctbp2) is an evolutionarily conserved transcriptional repressor that regulates fundamental processes such as cell proliferation and apoptosis. However, the potential role of Ctbp2 in cardiomyocyte proliferation and heart regeneration remains unclear. In this study, we aim to explore the important role of Ctbp2 in cardiomyocyte proliferation and the regeneration of injured adult hearts.

**Methods and results:**

In this study, we found that the expression of Ctbp2 in cardiomyocytes is downregulated after adulthood. Silencing Ctbp2 in cardiomyocytes on the post-natal day 1 (P1) reduced the proliferation ability of cardiomyocytes, whereas overexpressing Ctbp2 enhanced the proliferation ability of cardiomyocytes. Additionally, overexpressing Ctbp2 via adeno-associated virus-9 (AAV9) had no effect on the hearts of normal adult mice, but in the case of heart injury, overexpression of Ctbp2 in adult mice cardiomyocytes promoted cardiomyocyte proliferation. Mechanistically, the transcriptional repressor Ctbp2 acts as a metabolite sensor, and its regulation of cardiomyocyte proliferation is influenced by the metabolites NADH/NAD+ and fatty acyl-CoAs. Ctbp2 is activated by the intracellular accumulation of NADH during cardiomyocyte ischemia and hypoxia, inhibiting the transcriptional activity of the transcription factor FoxO1, thereby repressing the expression of the target genes and cell cycle negative regulators p21 and p27, allowing cardiomyocytes to re-enter the cell cycle. In contrast, normal adult cardiomyocytes mainly use fatty acid oxidation metabolism as their primary energy source, and the intracellular production of fatty acyl-CoAs inactivates Ctbp2, thus preventing it from inhibiting FoxO1 mediated cell cycle arrest.

**Conclusion:**

In conclusion, this study demonstrates that the Ctbp2-FoxO1-p21/p27 axis can promote cardiomyocyte proliferation and heart regeneration. As a metabolite sensor, Ctbp2 is activated during cardiomyocyte ischemia and hypoxia, while it is inactivated under normal conditions. This controllable and transient regulation of cardiomyocyte proliferation can avoid the detrimental effects on cardiac function caused by long-term regulation of cardiomyocyte proliferation, such as hypertrophic cardiomyopathy or heart failure. This provides new targets and new ideas for addressing the issues of cardiomyocyte proliferation and heart regeneration.

**Supplementary Information:**

The online version contains supplementary material available at 10.1186/s10020-025-01168-8.

## Introduction

Heart diseases, including myocardial infarction, are the leading causes of death worldwide (Bassat et al. 2017; Novoyatleva et al. 2010; Shapiro et al. 2014). The adult mammalian heart is one of the tissues with the poorest regenerative capacity among adult tissues, and it cannot regenerate after injury. Therefore, improving the heart’s self-repair capacity has become a global challenge that scientists are focusing on. In contrast, the hearts of neonatal mammals have a unique regenerative capacity after various types of injury. In mice, this regenerative ability is lost by the seventh day after birth (Cui et al. 2020; Han et al. 2021; Wang et al. 2020). The regenerating cardiomyocytes in the neonatal heart do not originate from cardiac stem cells but from the proliferation of pre-existing cardiomyocytes. After birth, the cell cycle-related genes in cardiomyocytes are gradually repressed during development. A key feature of the neonatal heart’s regenerative response is the activation of cardiomyocyte proliferation after injury, but adult cardiomyocytes fail to reactivate the neonatal proliferation-related transcriptional network after myocardial infarction. Therefore, reactivating the dormant neonatal heart regenerative response in adulthood to re-initiate the cardiomyocyte cell cycle might be a therapeutic approach for heart repair, but it requires a deeper understanding of the regeneration mechanisms (Porrello et al. 2011, 2013). Significant progress has been made in this research field in recent years. For example, The Hippo signaling pathway has been proven to be a key signaling pathway regulating the proliferation and differentiation of cardiomyocytes, playing a crucial role in heart development and regeneration (Wang et al. 2018b). The James Martin laboratory at Baylor College of Medicine was the first to discover that knocking out the Hippo signaling pathway during mouse embryonic development promotes cardiomyocyte proliferation, leading to cardiac hypertrophy (Heallen et al. 2011). Subsequent research in the laboratory has found that knocking out the Hippo signaling pathway can activate the proliferation ability of adult mouse cardiomyocytes and promote repair after heart injury (Heallen et al. 2013; Leach et al. 2017). In addition, knocking down the Hippo signaling pathway through gene therapy can repair damaged pig cardiomyocytes, reduce scar size, and improve cardiac function (Liu et al. 2021). The Eric Olson laboratory at the University of Texas Southwestern Medical Center found that the downstream effector protein YAP of the Hippo signaling pathway, which is specifically deficient in the heart, hinders neonatal heart regeneration. Conversely, overexpression of YAP in adult hearts can stimulate heart regeneration and improve heart function after myocardial infarction (Xin et al. 2013). These studies indicate that knocking out the Hippo signaling pathway has enormous therapeutic potential for treating heart damage. In addition, Eric Olson’s laboratory has created the most complete database of neonatal mouse heart regeneration in history. Ccl24, which encode cytokines, and mRNA binding protein Igf2bp3, have been identified as previously unrecognized regulators of cardiomyocyte proliferation. This study provides new insights into the molecular basis of neonatal heart regeneration and identifies novel genes that promote heart regeneration (Wang et al. 2019). Certain long non-coding RNAs are differentially expressed in neonatal and adult mice cardiomyocytes and can regulate cardiomyocyte proliferation and heart regeneration (Cai et al. 2018). Oxygen levels in the heart are another key regulator of cardiomyocyte proliferation following ischemic injury (Nakada et al. 2016). However, the molecular mechanisms that regulate the cardiomyocyte cell cycle are largely unknown, and further exploration of heart regeneration targets is needed. Long-term regulation of these pathways may lead to hypertrophic cardiomyopathy or heart failure, for example, sustained inactivation of the Hippo signaling pathway can result in extensive cardiomyocyte dedifferentiation, compromising cardiac function and the repair of the damaged heart after myocardial infarction (D’Uva et al. 2015; Ikeda et al. 2019). Therefore, exploring controllable, transient methods to regulate these pathways to replace lost cardiomyocytes due to disease and improve myocardial function after injury is the starting point of this study.

Recent studies have revealed through sequencing that compared to adult mice cardiomyocytes, Ctbp2 is highly expressed in neonatal mice cardiomyocytes, suggesting its potential involvement in regulating cardiomyocyte proliferation and heart regeneration, which has piqued our interest (Quaife-Ryan et al. 2017). By the consulting expression abundance of Ctbp2 in various organs and cells of mice through BioGPS, we found that Ctbp2 is expressed in multiple organs (heart, kidney, lungs, etc.) and cells (embryonic stem cells, immune cells, etc.) of mice. However, we found that Ctbp2 has the highest expression abundance in embryonic stem cells, far higher than other tissues and cells. According to the GEO database (GSE50704, GSE51483, GSE93269), Ctbp2 is highly expressed in fetal heart and cardiomyocytes, and lowly expressed in adult heart and cardiomyocytes. Researchers in Eric Olson’s laboratory have stated that the regeneration process of the heart in newborn mice is a unique response, meaning that mice with myocardial damage can undergo myocardial regeneration because they still retain the animal’s embryonic cardiogenic gene program. This prompted us to initiate research on Ctbp2 in cardiomyocytes (Wang et al. 2019). The Ctbp family was initially discovered in adenovirus research, where it interacted with the C-terminus of the adenovirus E1A protein as a phosphorylated protein. There are two isoforms of Ctbp, Ctbp1 and Ctbp2. Ctbp2 contains a nuclear localization signal in its N-terminal region, indicating that its function is primarily related to transcription, while Ctbp1, lacking this signal, is distributed throughout the cell and primarily functions non-transcriptionally (Bergman et al. 2006; Liberali et al. 2008; Pagliuso et al. 2016). The main role of Ctbp2 is to regulate processes such as cell proliferation and apoptosis by inhibiting gene transcription (Guan et al. 2013; Ju et al. 2021; Zhang et al. 2015). Since Ctbp2 is a transcriptional repressor that lacks DNA-binding ability, it gains access to genomic regions by binding to its target transcription factors (Cell cycle related transcription factors: FoxO1, SOX6, E2F7, etc.), which then recruit chromatin-modifying enzymes to alter chromatin structure, thereby inhibiting gene transcription (Byun and Gardner 2013; Chinnadurai 2007; Sekiya et al. 2021; Murakami et al. 2001; Zhao et al. 2014; Liu et al. 2013). Given that the transcription factors regulated by Ctbp2 are mainly associated with the cell cycle, most studies on Ctbp2 have focused on tumor cells (Acosta-Baena et al. 2022). Research has shown that Ctbp2 plays a crucial role in the occurrence and development of tumors such as breast cancer, colorectal cancer, ovarian cancer, and prostate cancer (Guan et al. 2013; Sengupta et al. 2013; Zhao et al. 2019). Ctbp2 promotes epithelial-mesenchymal transition by inhibiting the expression of E-cadherin (Ma et al. 2019; Zhao et al. 2007). Additionally, it may act as a transcriptional corepressor, negatively regulating certain tumor suppressors, thus promoting tumorigenesis and development (Thio et al. 2004; Wang et al. 2018a). In addition, Ctbp2 is also involved in the regulation of development and metabolism. The Ctbp gene in fruit flies is essential for embryonic development, and embryos lacking Ctbp2 exhibit developmental defects (Nibu et al. 1998; Poortinga et al. 1998). Mouse embryos lacking Ctbp2 die in mid pregnancy and exhibit developmental defects in several tissues, including delayed development of the forebrain and hindbrain. The composite mutant lacking Ctbp1 and Ctbp2 exhibits a more severe embryonic phenotype, characterized by developmental defects in the heart and neuroectoderm, as well as earlier embryonic death (Hildebrand and Soriano 2002). According to reports, the affinity of Ctbp2 for NADH is 100 times higher than that for NAD+, indicating that Ctbp2 can sense the redox state of cells (Kim and Dang 2005). The latest research indicates that Ctbp2, as a key metabolite receptor, plays a crucial role in the pathogenesis of metabolic imbalance and obesity, suggesting the potential of targeting Ctbp2 for the treatment of metabolic diseases (Sekiya et al. 2021; Sekiya et al. 2024). However, the potential role of Ctbp2 in the regulation of cardiomyocyte proliferation and heart regeneration remains unclear.

In this study, we found that the expression level of Ctbp2 in adult mice cardiomyocytes decreased compared to neonatal mice cardiomyocytes. This decrease aligns with the gradually declining proliferative capacity of mice cardiomyocytes after birth, suggesting that Ctbp2 might play a role in regulating cardiomyocyte proliferation. Further investigation revealed that overexpressing Ctbp2 not only promoted the proliferation of primary neonatal mice cardiomyocytes but also enhanced cardiomyocyte proliferation following myocardial infarction in adult mice. However, it did not affect normal adult mice cardiomyocytes. Previous studies have indicated that during embryonic and neonatal stages, cardiomyocytes exhibit a higher dependence on glycolysis as an energy source, with mitochondrial oxidative metabolism being relatively underdeveloped. However, to meet the increased energy demand during postnatal heart development, a metabolic shift towards fatty acid oxidation occurs (Bae et al. 2021; Lopaschuk et al. 1992). Other research has shown that an imbalance between myocardial oxygen supply and demand can lead to the accumulation of NADH when mitochondrial oxygen utilization ceases (Harden et al. 1979). In our study, we discovered that NADH and the fatty acid oxidation product fatty acyl-CoAs in cardiomyocytes affect the ability of Ctbp2 to inhibit the transcriptional activity of transcription factor FoxO1. During myocardial ischemia and hypoxia, the intracellular accumulation of NADH activates Ctbp2, which inhibits the transcriptional activity of FoxO1. This inhibition suppresses the expression of cell cycle negative regulators p21 and p27, allowing cardiomyocytes to re-enter the cell cycle. Conversely, in normal adult cardiomyocytes, fatty acyl-CoAs produced during fatty acid oxidation inactivates Ctbp2, thereby preventing the inhibition of FoxO1 mediated cell cycle arrest.

Ctbp2 acts as a metabolite sensor, becoming activated in cardiomyocytes during ischemia and hypoxia. This activation inhibits FoxO1 mediated cell cycle arrest and promotes cardiomyocyte proliferation. Under normal conditions, however, Ctbp2 remains inactive. This method of controllable, transient regulation of cardiomyocyte proliferation helps to prevent cardiac function impairment caused by hypertrophic cardiomyopathy or heart failure due to prolonged regulation of cell proliferation. It provides a new target and innovative approach for addressing challenges in cardiomyocyte proliferation and cardiac regeneration.

## Materials and methods

### Animals

C57BL/6 mice were purchased from Liaoning Changsheng Biotechnology (Liaoning, China). Individually raised in a temperature-controlled environment (22 ± 1 °C), with a light/dark cycle of 12 h, food and water can be freely obtained. The weight and physiological status of animals in different experimental groups are similar. All experimental animals were raised according to the Guidelines for the Care and Use of Experimental Animals issued by China National Health Research Institute, and the experimental protocol was approved by the Ethics Committee of Harbin Medical University.

### Primary cardiomyocyte isolation and culture

Quickly remove the hearts of neonatal mice and cut them into small pieces with scissors. Then, the cells were isolated and digested several times using 0.125% trypsin solution (Beyotime, China). Next, collect the supernatant after each round of digestion and transfer it to DMEM culture medium with 10% fetal bovine serum and 1% penicillin streptomycin solution added. Total cell suspension used 70 μM filter membrane filtration, centrifuge at 1500 rpm for 5 min. After removing the supernatant, the cells were cultured in DMEM medium with 10% fetal bovine serum and 1% penicillin streptomycin for 90 min. During this process, most non-CMs adhere to the culture bottle, while CMs remain suspended. Collect suspended cells and culture them at a certain density on a culture plate, and add Brdu (final concentration of 100 μmol/L) to the culture medium to inhibit the growth of fibroblasts.

### Quantitative RT-PCR

Total RNA was extracted from cell and tissue samples using Trizol reagent (TAKARA, Japan). Total RNA was dissolved in RNase-free water (DEPC) and reverse-transcribed to cDNA. Then, Hieff qPCR SYBR Green Master Mix (YEASEN, China) was used for quantitative RT-PCR. The primers are listed in Table 1.

**Table 1:** List of primer sequences used for qRT-PCR

Primer name	Sequence (5′ to 3′)
Ctbp2 fwd	TCAATCTGTATCGGCGGAACA
Ctbp2 rev	CCCAGAGATCGTTCTATCCCATC
FoxO1 fwd	GGGTCCCACAGCAACGATG
FoxO1 rev	CACCAGGGAATGCACGTCC
p21 fwd	CCTGGTGATGTCCGACCTG
p21 rev	CCATGAGCGCATCGCAATC
p27 fwd	TCTCTTCGGCCCGGTCAAT
p27 rev	AAATTCCACTTGCGCTGACTC
CDK1 fwd	AGAAGGTACTTACGGTGTGGT
CDK1 rev	GAGAGATTTCCCGAATTGCAGT
CDK4 fwd	AAGGTCACCCTAGTGTTTGAGC
CDK4 rev	CCGCTTAGAAACTGACGCATTAG
ccnb1 fwd	AAGGTGCCTGTGTGTGAACC
ccnb1 rev	GTCAGCCCCATCATCTGCG
ccnd1 fwd	GCGTACCCTGACACCAATCTC
ccnd1 rev	CTCCTCTTCGCACTTCTGCTC
18s fwd	AGTCCCTGCCCTTTCTACACA
18s rev	CGATCCGAGGGCCTCACTA

### Western blotting

The isolated mouse cardiomyocytes or heart tissue were lysed in a cold lysis buffer (Beyotime, China) containing Phenylmethanesulfonyl fluoride (PMSF), and the protein was quantified using the BCA protein analysis kit (Beyotime, China). Mice cardiomyocytes or mice heart tissue nucleus were separated using the cytoplasm/nucleus separation kit (BB-36021, Bestbio, China) and tissue cytoplasm/nucleus separation kit (BB-36022, Bestbio, China). The obtained nucleus were lysed using the above method and the proteins were quantified. Protein samples were separated by 10% sodium dodecyl sulfate–polyacrylamide gel electrophoresis (SDS-PAGE), and then transferred to nitrocellulose membrane. After blocking, incubate the membrane overnight with the following primary antibodies at 4 °C: anti-Ctbp2 (1:1000; ab128871, Abcam, UK), anti-FoxO1 (1:1000; 2880T, Cell Signaling Technology, USA), anti-p21 (1:1000; ab188224, Abcam, UK), GAPDH (1:1000, TA802519, OriGene, USA). Wash the membrane with Tris Buffered saline/0.1% Tween 20 (TBST) and incubate with secondary antibody at room temperature for 1 h.

### Cell transfection

Using transfection reagents, adenovirus was transfected into cardiomyocytes to silence and overexpress Ctbp2. Adenovirus and transfection reagents were designed and synthesized by Genechem (Shanghai, China), and the transfection methods were carried out according to the manufacturer’s instructions. The transfected cells were analyzed after 48 or 72 h.

### Immunofluorescence

Wash the culture medium with PBS for cells cultured in vitro. Then, fix the cells with 3% frozen paraformaldehyde. Frozen section fixes with cold acetone. Cell or tissue sections were permeabilized and blocked with 10% horse serum containing 0.3% Triton X-100 at room temperature for 1 h. Then, the cells or tissue sections were incubated overnight with the following primary antibodies at 4 °C: anti-cTnT (1:400; ab8295, Abcam, UK), anti-Ctbp2 (1:50; ab128871, Abcam, UK), anti-Ki67 (1:100; 14–5698-82, Invitrogen, USA), anti-pH3 (1:1000; ab303567, Abcam, UK). After washing the cell or tissue sections with PBS, incubate them with the following secondary antibodies at room temperature for 1 h: goat anti-mouse IgG/Alexa Fluid 488 (1:500; ab150113, Abcam, UK), donkey anti-rat IgG/Alexa Fluid 647 (1:500; ab150155, Abcam, UK), Goat anti-Rabbit IgG (H+L) Highly Cross-Adsorbed Secondary Antibody/Alexa Fluor Plus 647 (1:500; A32733TR, Invitrogen, USA). After washing the cell or tissue sections with PBS, incubate them with DAPI at room temperature. Use a fluorescence microscope for image acquisition.

### 5-Ethynyl-2′-deoxyuridine (EDU) staining assay

We used the EDU cell imaging kit (40277ES60, YEASEN, China) to detect cells proliferation. Briefly, add EDU (final concentration of 10 μmol/L) to the culture medium and co-incubate with cells 24 h in advance. Firstly, fix the cells with 4% paraformaldehyde for 15 min. Then permeate with 0.3% Triton X-100. Treat cells with EDU reaction mixture for 30 min, and stain the nucleus with DAPI. Use a fluorescence microscope for image acquisition.

### Infection of adeno-associated virus

The AAV9 vectors with the cardiomyocyte-specific cTnT promoter carrying Ctbp2 and negative control (NC) were constructed by Genechem (Shanghai, China). The AAV9 virus (1×1012v.g per mouse) was delivered into C57BL/6 male mice (4–6 weeks old) by tail vein injection for 3 weeks before MI surgery.

### Myocardial infarction (MI) mouse model

C57BL/6 male mice (8 weeks old) were anesthetized intraperitoneally with pentobarbital sodium and breathed using Small Animal Ventilators. After thoracotomy, ligate the left anterior descending branch of the coronary artery with 7-0 suture. The sham group mice underwent similar surgery without blocking the coronary artery. After the surgery, warm the mice until they wake up.

### Echocardiography

Use small animal cardiac ultrasound instrument Vevo3100LT (Visualsonics, JPN) to detect left ventricular systolic function in mice. The mice were anesthetized with pentobarbital sodium throughout the entire process for non-invasive examination. Using Vevo lab software, calculate the left ventricular ejection fraction (LVEF) and left ventricular fraction shortening (LVFS) based on the end diastolic and end systolic inner diameters measured by M-mode.

### 5-Bromodeoxyuridine (Brdu) staining

Mice were intraperitoneally injected with Brdu (10 mg/mL), 200 µL per mouse, for 7 consecutive days. Take heart tissue for frozen sectioning. Cold acetone fixed slicing for 15 min. After washing the tissue slices with PBS, incubate them at 37 °C for 30 min with 2 mol/L HCL. After washing the tissue slices with 0.1 mol/L boric acid buffer, 10% horse serum containing 0.3% Triton X-100 was blocked at room temperature for 1 h. Then, the tissue slices were incubated overnight with primary antibodies at 4 °C: anti-Brdu (1:200; ab6326, Abcam, UK). After washing the tissue slices with PBS, the secondary antibody was incubated at room temperature for 1 h. After washing the tissue slices with PBS, DAPI was incubated at room temperature. Use a fluorescence microscope for image acquisition.

### 2,3,5-Triphenyltetrazolium chloride (TTC) staining

Measure the infarct size of the heart using the TTC (T8877, sigma, USA). Remove the entire heart from the mouse body and rapidly freeze for 5–10 min. Cut the heart into 2 mm thick transverse slices and place them in a well plate containing 2% TTC solution. Incubate at 37 °C for 15–30 min. Finally, take photos of the slices and measure the infarct area of different slices using ImageJ software.

### Wheat germ agglutinin (WGA) staining

Frozen sections of mice heart tissue were fixed with cold acetone for 15 min. After washing the tissue slices with PBS, the tissue slices were incubated with 5 μg/mL WGA (L4895, sigma, USA) at room temperature for 10 min. After washing the tissue slices with PBS, incubate them with DAPI at room temperature for 8 min. Use a fluorescence microscope for image acquisition. ImageJ software measures the cross-sectional area of myocardial cells.

### Masson’s trichrome staining

For paraffin sections, heart tissue was fixed with 4% PFA and subsequently sent to AOPENG Biotechnology (Shanghai, China) for Masson’s trichrome staining. Briefly, the tissue was embedded in paraffin, sliced, and dehydrated. The paraffin sections were then stained using Masson’s staining solution (AOPENG, China) according to the protocol provided by the company’s technical personnel. Microscopic examination, image acquisition, and analysis were performed. For frozen sections, tissue sections were fixed with cold acetone for 15 min, washed with distilled water, and stained using Masson’s trichrome stain kit (G1340, Solarbio, China). Specific methods were followed as described in the reagent kit instructions. Microscopic examination, image acquisition, and analysis were conducted.

### Sirius red staining

Frozen tissue sections were fixed with cold acetone for 15 min, washed with distilled water, and stained using the Sirius red stain kit (G1472, Solarbio, China). The staining procedure was performed according to the instructions provided with the reagent kit. Microscopic examination, image acquisition, and analysis were carried out.

### Cell counting kit-8

After overexpression of Ctbp2 in primary neonatal mice cardiomyocytes, different concentrations of KA, different ratios of lactate, and different concentrations of sodium palmitate were added to the cell culture medium. At the same time, 1/10 volume of CCK8 solution (C0005, TargetMol, USA) was added, thoroughly mixed, and then placed in a cell culture incubator for cultivation. The absorbance (450 nm) was measured every hour using microplate reader.

### Nuclear NADH/NAD+ and fatty acyl-CoA content

After euthanizing SHAM mice and myocardial infarction mice (3 days after myocardial infarction), the mouse heart tissue was isolated, excess tissues such as large blood vessels were removed, and the heart tissue was washed in cold physiological saline. After washing, it was dipped to dry on filter paper. Place the mouse heart in a pre-cooled mouse heart slicing mold, observe the heart infarction under a stereomicroscope, divide the heart into zones (white for infarction area, pink for border, red for remote), and use a sharp blade for segmentation. After cutting the heart tissue into pieces, the tissue cytoplasm/nucleus separation kit (BB-36022, Bestbio, China) was used to extract the nucleus. NAD+/NADH assay kit (S0175, Beyotime, China) was used to detect the levels of NADH and NAD+ in the nucleus through the WST-8 colorimetric method. The extraction and detection of fatty acyl-CoA were based on the method described in the previous research (Sekiya et al. 2021; Mangino et al. 1992). The specific method is to separate lipids from the nucleus using the Bligh Dyer method, while collecting fatty acyl-CoA distributed in methanol/aqueous phase. Fatty acyl-CoA is oxidized by fatty acyl-CoA oxidase (A0270, Solarbio, China) to produce 2,3-trans-enyl-CoA and H_2_O_2_. Indirect quantification of fatty acyl-CoA by detecting H_2_O_2_ content using the H_2_O_2_ content assay kit (BC3595, Solarbio, China).

### Statistical analysis

All results were analyzed using GraphPad Prism 8. All data were expressed as the mean ± SD. Unpaired Student’s t-test was used for two-group comparisons and one-way analysis of variance (ANOVA) was used for multigroup comparisons. *P* < 0.05 was considered statistically significant:*P < 0.05; **P < 0.01; ***P < 0.001.

## Results

### Ctbp2 participates in the regulation of cardiomyocytes proliferation

**Fig. 1:**
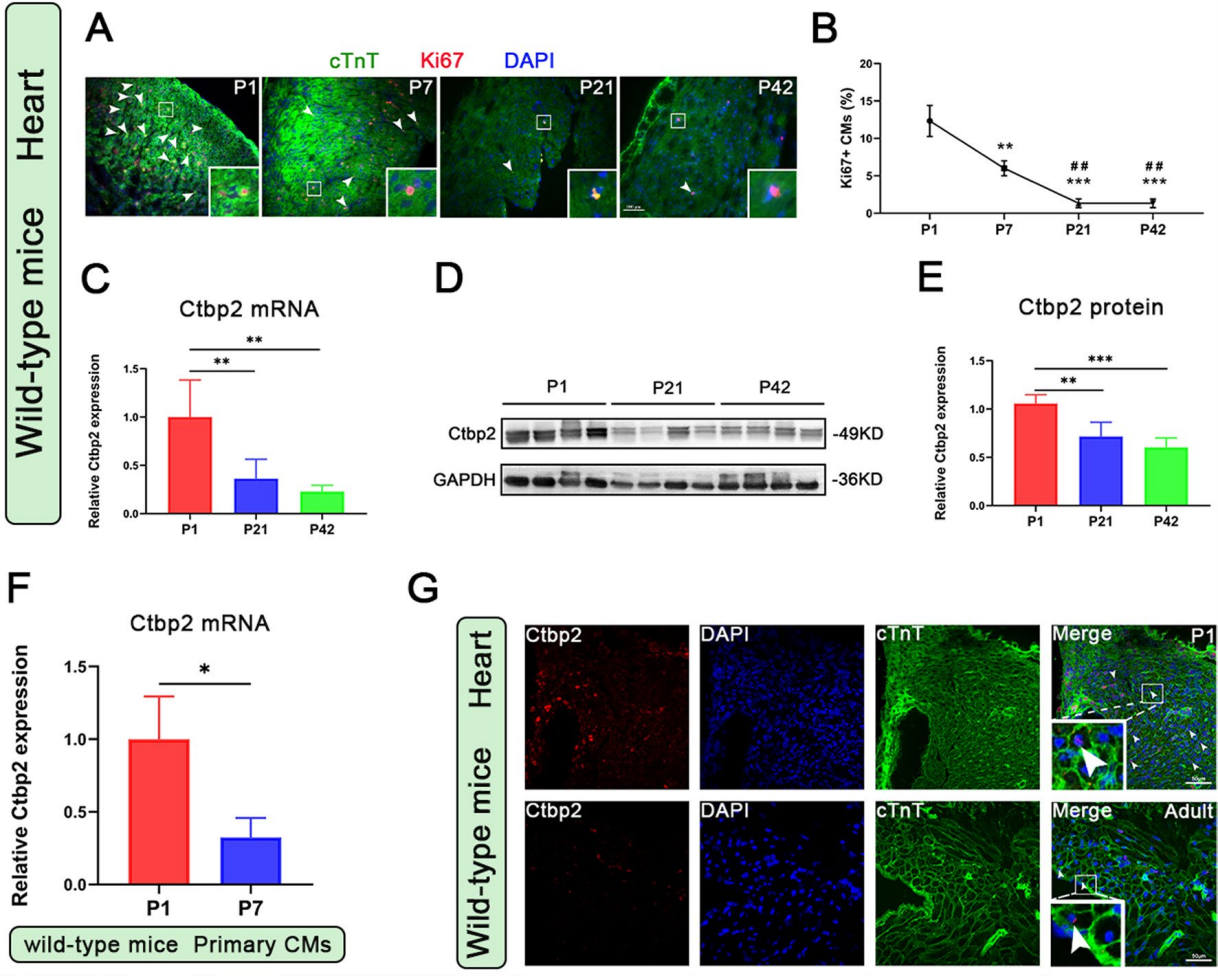
Ctbp2 participates in the regulation of cardiomyocytes proliferation. **A** Ki67 and cTnT immunofluorescence staining of heart from P1, P7, P21, and P42 mice. Scale bar = 100 µm. **B** Statistical analysis of the proportion of Ki67-positive cardiomyocytes (n = 3). Data were expressed as mean ± SD. *p vs. the P1 group, ^#^*p* vs. the P7 group. **P < 0.01, ***P < 0.001, ^##^*P* < 0.01, One-way ANOVA. **C** qPCR analysis of Ctbp2 in hearts from P1, P21, and P42 mice (n ≥ 4). Data were expressed as mean ± SD. **P < 0.01, One-way ANOVA. **D**, **E** Western-blot analysis of Ctbp2 in hearts from P1, P21, and P42 mice (n = 4). Data were expressed as mean ± SD. **P < 0.01, ***P < 0.001, One-way ANOVA. **F** qPCR analysis of Ctbp2 in cardiomyocytes from P1 and P7 mice (n = 3). Data were expressed as mean ± SD. *P < 0.05, t test. **G** Ctbp2 and WGA immunofluorescence staining of heart from P1 and Adult mice. Scale bar = 50 µm

First, we examined the changes in cardiomyocyte proliferative capacity at different developmental stages in mice. Using Ki67 and cardiac troponin T (cTnT) immunofluorescence staining, we assessed the proliferative capacity of cardiomyocytes in P1, P7, P21, and P42 mice heart tissues. The results showed a rapid decline in the proliferative capacity of cardiomyocytes over time after birth (Fig. 1A, B). Next, we analyzed the expression levels of Ctbp2 in P1, P21, and P42 mice heart tissues at both the mRNA and protein levels. The results indicated that the expression level of Ctbp2 in adult mice heart decreased compared to that in P1 mice (Fig. 1C–E). Then, using trypsin digestion, we extracted cardiomyocytes from P1 and P7 mice heart tissues and analyzed the expression levels of Ctbp2 in these cells at the mRNA level. The results showed that the expression level of Ctbp2 in P7 cardiomyocytes was lower than that in P1 cardiomyocytes (Fig. 1F). Subsequently, we used Ctbp2 and WGA/cTnT immunofluorescence staining to detect the expression levels of Ctbp2 in cardiomyocytes in P1 and adult mice heart tissues. The results indicated that the expression level of Ctbp2 in adult cardiomyocytes was lower than that in P1 cardiomyocytes (Figs. 1G, S1). In summary, the expression level of Ctbp2 in adult cardiomyocytes decreased compared to P1 cardiomyocytes, consistent with the decline in the proliferative capacity of cardiomyocytes. This suggests that Ctbp2 may be involved in the regulation of cardiomyocyte proliferation in mice.

### Ctbp2 promotes the proliferation of primary neonatal mice cardiomyocytes

**Fig. 2:**
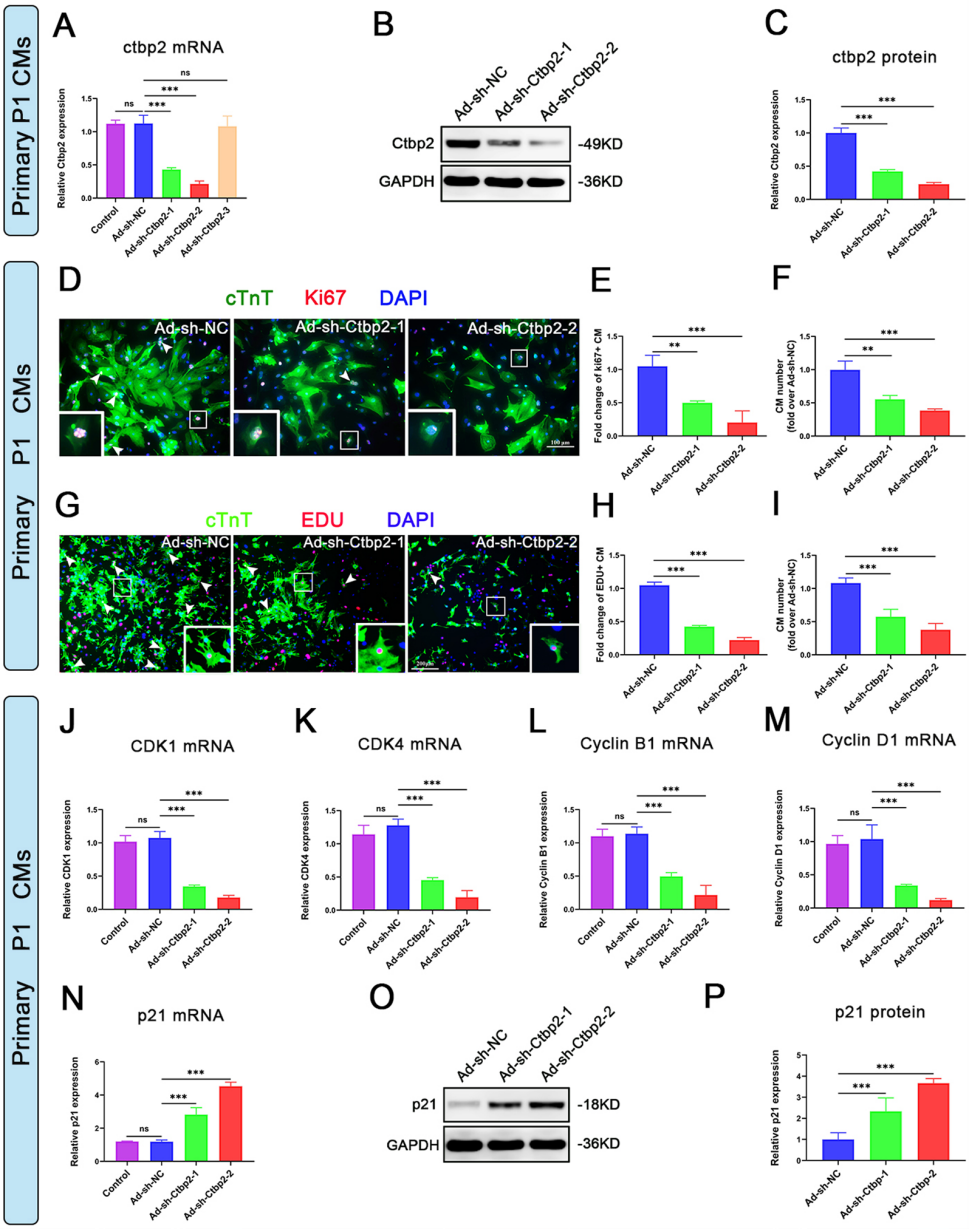
Silencing Ctbp2 inhibits the proliferation of primary neonatal mice cardiomyocytes. **A** qPCR analysis of Ctbp2 in cardiomyocytes from P1 mice after 72 h of transfection with Ad-sh-NC and Ad-sh-Ctbp2 (n ≥ 3). Data were expressed as mean ± SD. ***P < 0.001, One-way ANOVA. **B**, **C** Western-blot analysis of Ctbp2 in cardiomyocytes from P1 mice after 72 h of transfection with Ad-sh-NC and Ad-sh-Ctbp2 (n ≥ 3). Data were expressed as mean ± SD. ***P < 0.001, One-way ANOVA. **D** Ki67 and cTnT immunofluorescence staining of cardiomyocytes from P1 mice after 72 h of transfection with Ad-sh-NC and Ad-sh-Ctbp2. Scale bar = 100 µm. **E**, **F** Statistical analysis of the number of Ki67-positive cardiomyocytes and the total number of cardiomyocytes (n = 3). Data were expressed as mean ± SD. **P < 0.01, ***P < 0.001, One-way ANOVA. **G** EDU and cTnT immunofluorescence staining of cardiomyocytes from P1 mice after 72 h of transfection with Ad-sh-NC and Ad-sh-Ctbp2. Scale bar = 200 µm. **H**, **I** Statistical analysis of the number of EDU-positive cardiomyocytes and the total number of cardiomyocytes. Data were expressed as mean ± SD (n ≥ 3). ***P < 0.001, One-way ANOVA. **J**–**M** qPCR analysis of cell cycle activators (CDK1, CDK4, Cyclin B1 and Cyclin D1) in cardiomyocytes from P1 mice after 72 h of transfection with Ad-sh-NC and Ad-sh-Ctbp2 (n ≥ 3). Data were expressed as mean ± SD. ***P < 0.001, One-way ANOVA. **N** qPCR analysis of negative cell cycle regulators (p21) in cardiomyocytes from P1 mice after 72 h of transfection with Ad-sh-NC and Ad-sh-Ctbp2 (n = 3). Data were expressed as mean ± SD. ***P < 0.001, One-way ANOVA. **O**, **P** Western-blot analysis of negative cell cycle regulators (p21) in cardiomyocytes from P1 mice after 72 h of transfection with Ad-sh-NC and Ad-sh-Ctbp2 (n ≥ 3). Data were expressed as mean ± SD. ***P < 0.001, One-way ANOVA

To investigate the role of Ctbp2 in cardiomyocyte proliferation, we silenced or overexpressed Ctbp2 in P1 mice cardiomyocytes using adenoviral transfection. We validated the effects of adenoviral silencing or overexpression of Ctbp2 at the mRNA and protein levels (Figs. 2A–C, S2A–C). After silencing or overexpressing Ctbp2 in P1 cardiomyocytes, we assessed cardiomyocyte proliferation using Ki67 and 5-ethynyl-2′-deoxyuridine (EDU) immunofluorescence staining. The results showed that silencing Ctbp2 reduced the number of proliferating cardiomyocytes and total cardiomyocytes (Fig. 2D–I), while overexpressing Ctbp2 increased both populations (Fig. S2D–I). Subsequently, we performed qPCR to detect the expression of cell cycle activators (CDK1, CDK4, Cyclin B1, and Cyclin D1) in cardiomyocytes. Silencing Ctbp2 reduced the expression of CDK1, CDK4, Cyclin B1, and Cyclin D1 compared to the control (Fig. 2J–M), whereas overexpressing Ctbp2 increased their expression (Fig. S2J–M). Additionally, qPCR and Western blot were used to detect the expression of the cell cycle negative regulator p21 at the mRNA and protein levels. Silencing Ctbp2 increased the expression of p21 compared to the control (Fig. 2N–P), while overexpressing Ctbp2 decreased its expression (Fig. S2N–P). These results suggest that Ctbp2 promotes the proliferation of primary neonatal mice cardiomyocytes.

### Overexpression of Ctbp2 has no effect on the heart of normal adult mice

**Fig. 3:**
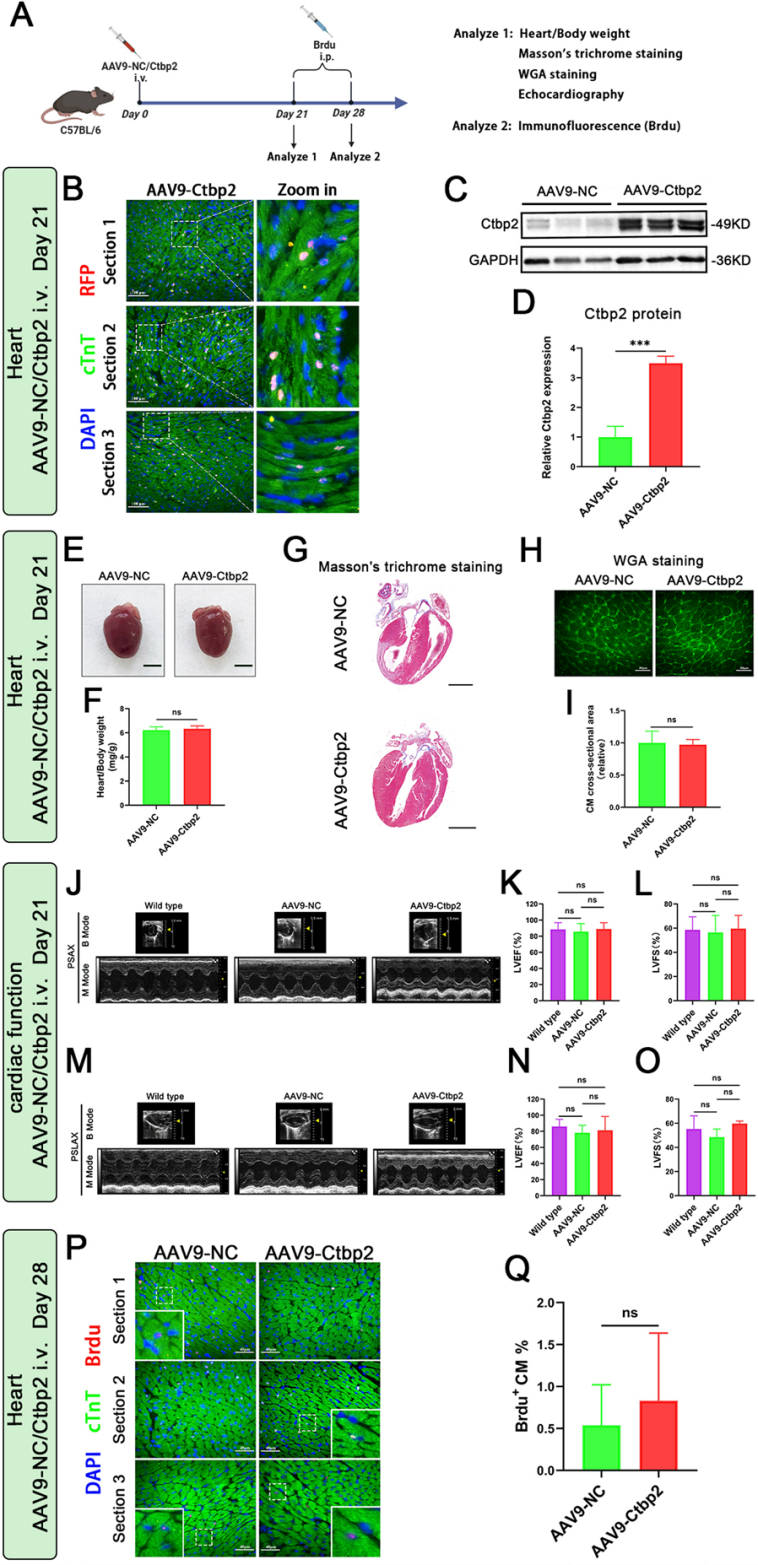
Overexpression of Ctbp2 has no effect on the heart of normal adult mice. **A** Schematic diagram for experimental procedure. **B** cTnT immunofluorescence staining of heart from 4 to 6-week-old mice after 21 days of tail vein injection of AAV9-cTnT-Ctbp2-RFP. Scale bar = 100 µm. **C**, **D** Western-blot analysis of Ctbp2 in hearts from AAV9-NC mice and AAV9-Ctbp2 mice (n = 3). Data were expressed as mean ± SD. ***P < 0.001, t test. **E** Representative images of heart from AAV9-NC mice and AAV9-Ctbp2 mice. Scale bar = 2 mm. **F** The ration of heart weight to body weight from AAV9-NC mice and AAV9-Ctbp2 mice (n = 5). Data were expressed as mean ± SD. t test. **G** Masson’s trichrome staining of heart from AAV9-NC mice and AAV9-Ctbp2 mice. Scale bar = 2 mm. **H**, **I** WGA staining of heart from AAV9-NC mice and AAV9-Ctbp2 mice, ImageJ measurement of cardiomyocyte size (n = 4). Scale bar = 40 µm. Data were expressed as mean ± SD. t test. **J**–**L** Cardiac function (PSAX) of wild-type mice, AAV9-NC mice and AAV9-Ctbp2 mice (n ≥ 3). Data were expressed as mean ± SD. One-way ANOVA. **M**–**O** Cardiac function (PSLAX) of wild-type mice, AAV9-NC mice and AAV9-Ctbp2 mice (n ≥ 3). Data were expressed as mean ± SD. One-way ANOVA. **P** Brdu and cTnT immunofluorescence staining of heart from AAV9-NC mice and AAV9-Ctbp2 mice. Scale bar = 40 µm. **Q** Statistical analysis of the proportion of Brdu-positive cardiomyocytes (n = 3). Data were expressed as mean ± SD. t test

To investigate whether Ctbp2 promotes adult cardiomyocyte proliferation, we performed the following experiment (Fig. 3A). First, we used 4–6 weeks old C57BL/6 mice and administered AAV9 carrying a cardiomyocyte-specific promoter cTnT via tail vein injection to achieve cardiomyocyte specific overexpression of Ctbp2. After 21 days of injection, immunofluorescence and Western blot confirmed that the injection of AAV9-cTnT-Ctbp2-RFP successfully overexpressed Ctbp2 in adult mice cardiomyocytes (Fig. 3B–D). Subsequent analysis of heart shape and the ratio of heart weight to body weight revealed that, compared to the control group, overexpression of Ctbp2 did not alter heart volume or weight in mice (Fig. 3E, F). Masson’s trichrome staining of mice heart tissue paraffin sections showed no fibrosis compared to the control group, and panoramic scanning of heart tissue sections indicated no structural changes following Ctbp2 overexpression (Fig. 3G). Wheat germ agglutinin (WGA) staining of frozen sections of mice heart tissue showed that the cross-sectional area of cardiomyocytes did not change, indicating no occurrence of cardiomyocyte hypertrophy compared to the control group (Fig. 3H, I). Echocardiography results indicated no significant changes in cardiac cavity size or structure on B-mode in both short-axis (PSAX) and long-axis (PSLAX) views after overexpression of Ctbp2. Additionally, M-mode showed no significant difference in left ventricular ejection fraction (LVEF) and left ventricular fraction shortening (LVFS) among the three groups, suggesting no significant differences in left ventricular systolic function (Fig. 3J–O). Mice were intraperitoneally injected with 5-bromodeoxyuridine (Brdu) for 7 consecutive days, and heart tissue was then collected for frozen sectioning. Brdu immunofluorescence staining revealed no difference in cardiomyocyte proliferation compared to the control group, indicating that overexpression of Ctbp2 did not affect cardiomyocyte proliferation (Fig. 3P, Q). Overall, these results suggest that under normal conditions, cardiomyocyte specific overexpression of Ctbp2 in adult mice has no impact on heart volume, weight, structure, function, cardiomyocyte cross-sectional area, or proliferative capacity.

### Overexpression of Ctbp2 has a protective effect on adult mice with heart injury

**Fig. 4:**
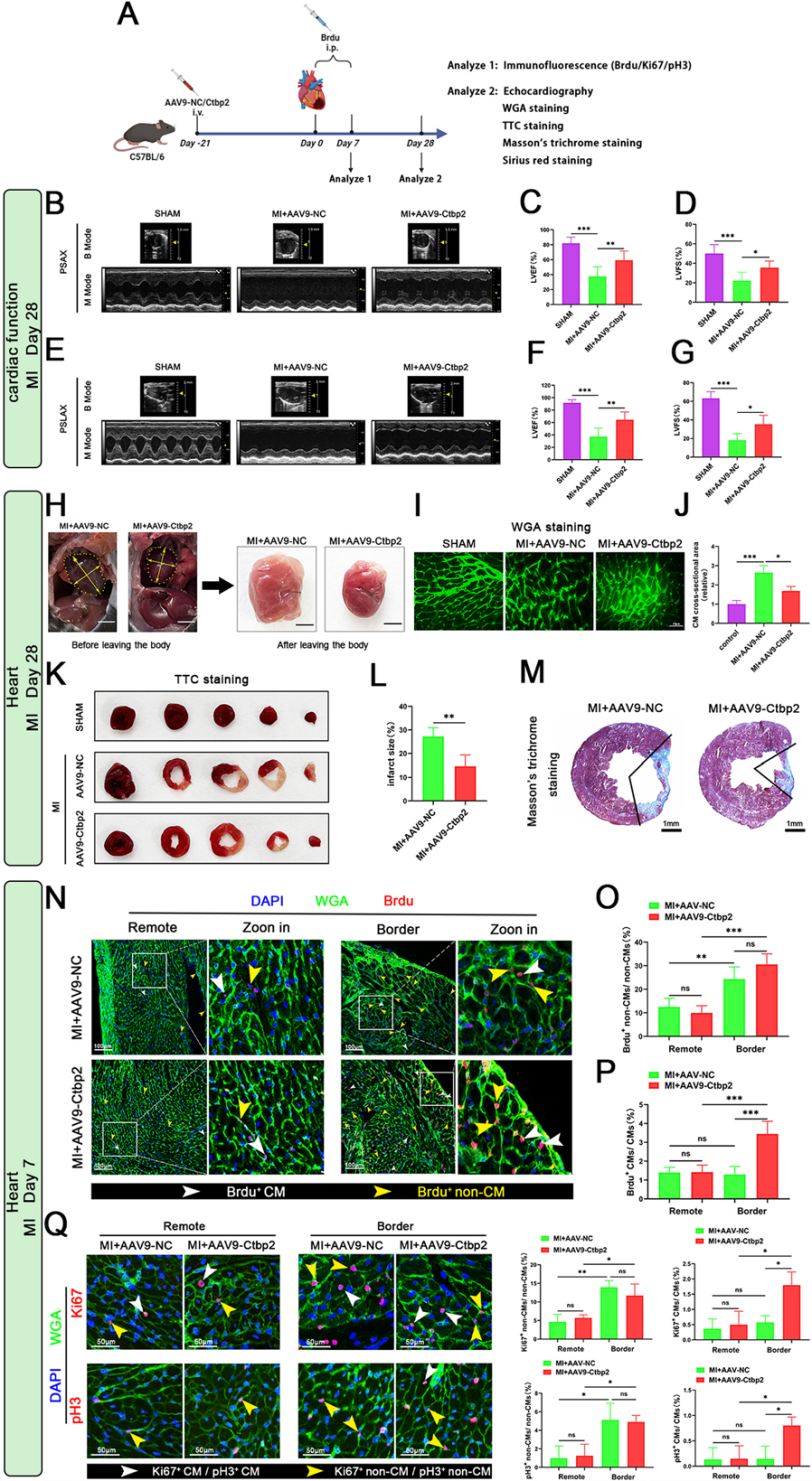
Overexpression of Ctbp2 has a protective effect on adult mice with heart injury. **A** Schematic diagram for experimental procedure. **B**–**D** Cardiac function (PSAX) of SHAM mice, MI+AAV9-NC mice and AAV9-Ctbp2 mice (n ≥ 5). Data were expressed as mean ± SD. *P < 0.05, **P < 0.01, ***P < 0.001, One-way ANOVA. **E**–**G** Cardiac function (PSLAX) of SHAM mice, MI+AAV9-NC mice and AAV9-Ctbp2 mice (n ≥ 4). Data were expressed as mean ± SD. *P < 0.05, **P < 0.01, ***P < 0.001, One-way ANOVA. **H** Representative images of heart from MI+AAV9-NC mice and AAV9-Ctbp2 mice. Scale bar = 4 mm (left), Scale bar = 2 mm (right). **I**, **J** WGA staining of heart from SHAM mice, MI+AAV9-NC mice and MI+AAV9-Ctbp2 mice, ImageJ measurement of cardiomyocyte size (n = 4). Scale bar = 40 µm. Data were expressed as mean ± SD. *P < 0.05, ***P < 0.001, One-way ANOVA. **K**, **L** TTC staining of heart from SHAM mice, MI+AAV9-NC mice and MI+AAV9-Ctbp2 mice (n = 4). Data were expressed as mean ± SD. **P < 0.01, t test. **M** Masson’s trichrome staining of heart from MI+AAV9-NC mice and MI+AAV9-Ctbp2 mice. Scale bar = 1 mm. **N** Brdu and WGA immunofluorescence staining of heart from MI+AAV9-NC mice and MI+AAV9-Ctbp2 mice. Scale bar = 100 µm. **O**, **P** Statistical analysis of the proportion of Brdu-positive non-cardiomyocytes and Brdu-positive cardiomyocytes (n ≥ 5). Data were expressed as mean ± SD. **P < 0.01, ***P < 0.001, One-way ANOVA. **Q** Ki67/pH3 and WGA immunofluorescence staining of heart from MI+AAV9-NC mice and MI+AAV9-Ctbp2 mice. Scale bar = 50 µm. Statistical analysis of the proportion of Brdu-positive non-cardiomyocytes and Brdu-positive cardiomyocytes (n = 3). Data were expressed as mean ± SD. *P < 0.05, **P < 0.01, One-way ANOVA

To further investigate the impact of Ctbp2 overexpression on the proliferative and regenerative capacity of adult cardiomyocytes, we constructed a mouse myocardial infarction model by ligating the left anterior descending coronary artery in mice with Ctbp2-overexpressing cardiomyocytes. We then evaluated cardiac function, cardiac hypertrophy, infarct size, fibrotic area, and cardiomyocyte proliferation following infarction (Fig. 4A). First, echocardiography showed no significant difference in initial cardiac function among the wild-type mice, AAV9-NC mice and AAV9-Ctbp2 mice before myocardial infarction(Fig. 3J–O), but overexpression of Ctbp2 significantly improved cardiac function after myocardial infarction (Fig. 4B–G). Observations of the heart before and after isolation showed that Ctbp2 overexpression significantly ameliorated cardiac deformation and hypertrophy post-myocardial infarction (Fig. 4H). Additionally, WGA staining of heart tissue frozen sections demonstrated that Ctbp2 overexpression significantly reduced the cross-sectional area of cardiomyocytes after myocardial infarction (Fig. 4I, J). 2,3,5-Triphenyltetrazolium Chloride (TTC) staining, Masson’s trichrome staining, and Sirius red staining of heart tissue further revealed that Ctbp2 overexpression significantly reduced the infarct size and fibrotic area (Figs. 4K–M, S3A). In adult mice with cardiomyocyte specific Ctbp2 overexpression, following the construction of myocardial infarction, Brdu was administered via intraperitoneal injection for 7 consecutive days. Brdu immunofluorescence staining of heart tissue was then used to assess cardiomyocyte proliferation. To investigate changes in proliferative capacity more precisely, we divided the infarcted heart tissue into infarction, border, and remote area (Fig. S3B). Meanwhile, we conducted statistical analysis on both cardiomyocytes and non-cardiomyocytes in the heart tissue (Fig. S3C). Brdu immunofluorescence staining results showed no significant difference in the percentage of Brdu positive non-cardiomyocytes and cardiomyocytes between MI+AAV9-NC and MI+AAV9-Ctbp2 mice in the remote area. However, in the border area, compared to MI+AAV9-NC mice, MI+AAV9-Ctbp2 mice showed no significant change in the percentage of Brdu positive non-cardiomyocytes but a significant increase in the percentage of Brdu positive cardiomyocytes (Figs. 4N–P, S3D–G). In addition, Ki67 and pH3 immunofluorescence staining also obtained the same results as Brdu immunofluorescence staining (Fig. 4Q). In summary, in adult mice, Ctbp2 overexpression promotes heart regeneration after myocardial infarction by stimulating cardiomyocytes to re-enter the cell cycle, thereby providing a protective effect against cardiac injury.

### Ctbp2 regulation of cardiomyocyte proliferation is influenced by the metabolites NADH/NAD+ and fatty acyl-CoAs

**Fig. 5:**
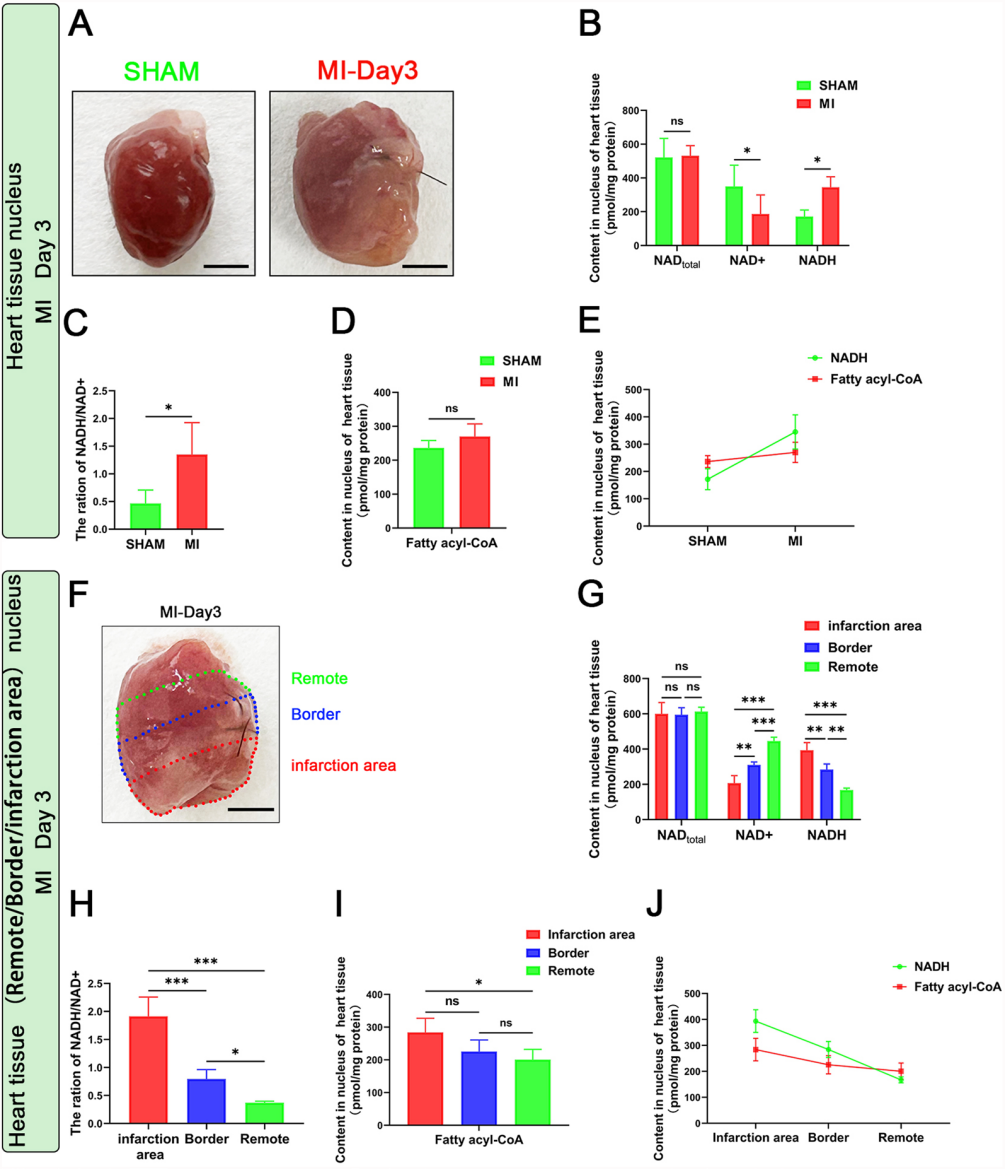
Content of nuclear metabolites NADH/NAD+ and fatty acyl-CoAs in heart tissue of mice. **A**–**E** NADH/NAD+ content and fatty acyl-CoAs content in heart tissue nucleus of SHAM mice and MI mice (n ≥ 3). Scale bar = 2 mm. Data were expressed as mean ± SD. *P < 0.05, **P < 0.01, t test. **F**–**J** NADH/NAD+ content and fatty acyl-CoAs content in heart tissue (infarction area, Border, Remote) nucleus of MI mice (n ≥ 3). Scale bar = 2 mm. Data were expressed as mean ± SD. *P < 0.05, **P < 0.01, ***P < 0.001, One-way ANOVA

These results indicate that under normal conditions, overexpression of Ctbp2 in adult mice cardiomyocytes does not affect their proliferative capacity. However, in the context of heart injury, overexpression of Ctbp2 in adult mice cardiomyocytes promotes their proliferation. Therefore, we aimed to explore why Ctbp2 overexpression in adult mice cardiomyocytes promotes proliferation only when heart injury is present. Previous studies have shown that in the liver, Ctbp2 can bind to the transcription factor FoxO1 and directly inhibit FoxO1 mediated gluconeogenesis. This inhibitory ability, however, is influenced by the metabolites NADH/NAD+ and fatty acyl-CoAs. NADH activates Ctbp2 by binding to it, enhancing its ability to inhibit FoxO1’s transcriptional activity, whereas fatty acyl-CoAs inactivates Ctbp2 by binding to it, thus inhibiting its transcriptional repression of FoxO1 (Sekiya et al. 2021, 2024). Other studies have indicated that transcription factor FoxO1, besides regulating metabolism, can also inhibit the cardiomyocyte cell cycle through various mechanisms, including the activation of its target genes, cell cycle negative regulators p21 and p27 (Evans-Anderson et al. 2008; Ho et al. 2008; Sengupta et al. 2013). It is well known that normal adult cardiomyocytes primarily rely on fatty acid oxidation for energy. However, during cardiomyocyte ischemia, the inability of mitochondria to utilize oxygen disrupts the oxidation of NADH to NAD+, leading to an accumulation of NADH within the cells. Given that Ctbp2, as a transcriptional repressor, is primarily localized in the nucleus, we first examined the changes in nuclear levels of NADH and fatty acyl-CoAs, an intermediate product of fatty acid oxidation, in cardiomyocytes under ischemic and hypoxic conditions. The results showed that in sham mice heart tissue, nuclear fatty acyl-CoAs levels were higher than NADH levels. However, in myocardial infarction mice heart tissue, nuclear NADH levels increased, NAD+ levels decreased, and the NADH/NAD+ ratio increased, with a slight increase in fatty acyl-CoAs levels. Consequently, nuclear NADH levels were higher than fatty acyl-CoAs levels in myocardial infarction mice heart tissue (Fig. 5A–E). Additionally, regional analysis of the infarcted heart tissue revealed that the closer the tissue was to the infarction area, the higher the NADH levels, the lower the NAD+ levels, and the higher the NADH/NAD+ ratio. Moreover, the closer the tissue was to the infarction area, the slightly higher the fatty acyl-CoAs levels. Therefore, in the remote area, nuclear fatty acyl-CoAs levels were higher than NADH levels, whereas in the border area and infarction area, nuclear NADH levels were higher than fatty acyl-CoAs levels (Fig. 5F–J). Based on previous research and the above experimental results, we hypothesize that in normal adult cardiomyocytes, fatty acid oxidation predominates as the primary energy source, with the intermediate product fatty acyl-CoAs inhibiting Ctbp2 activity and preventing it from exerting its effects. However, during ischemia and hypoxia, the accumulation of NADH within the cells activates Ctbp2, enabling it to promote cardiomyocyte proliferation.

**Fig. 6:**
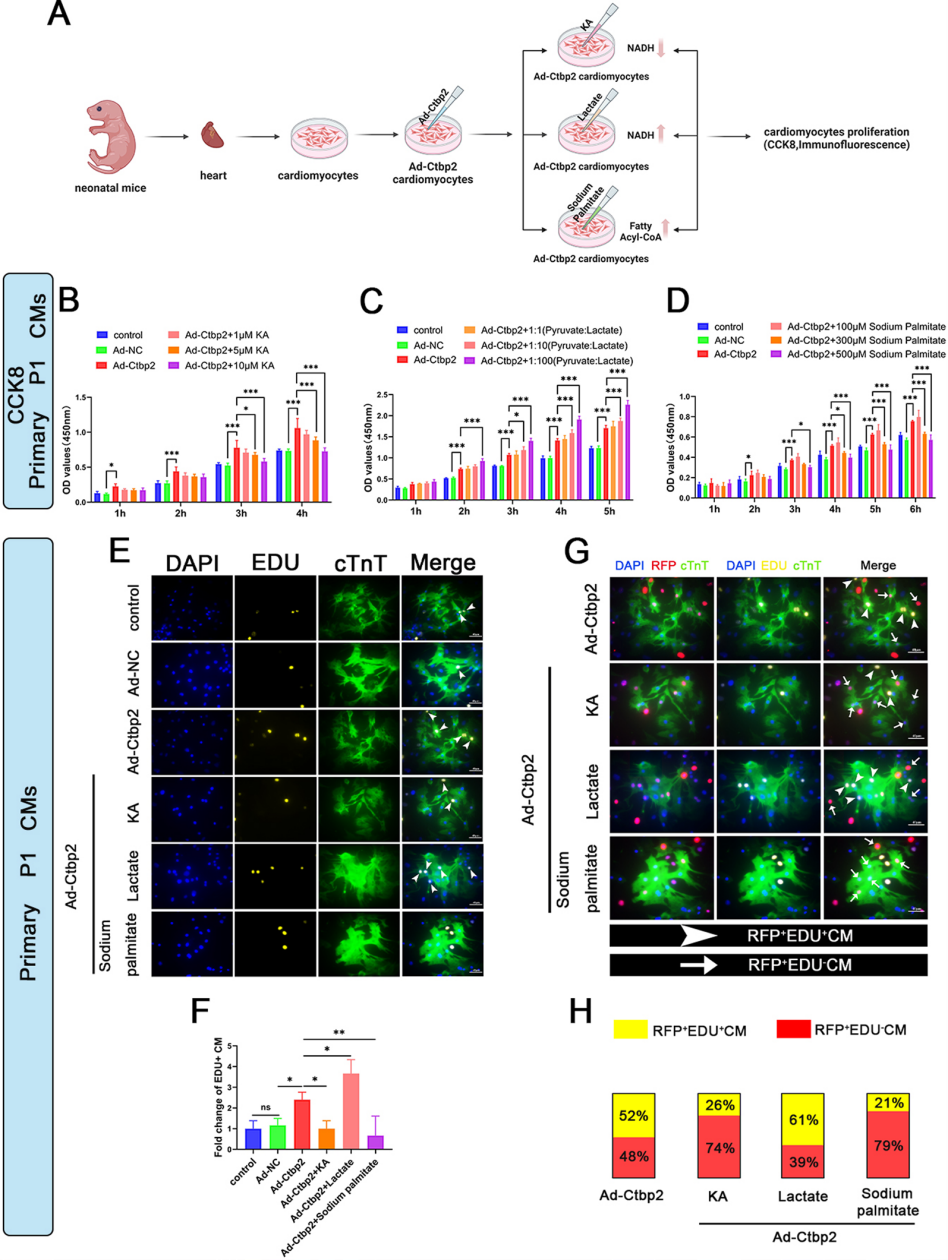
Ctbp2 regulation of cardiomyocyte proliferation is influenced by the metabolites NADH/NAD+ and fatty acyl-CoAs. **A** Schematic diagram for experimental procedure. **B** CCK8 analysis of control, Ad-NC, Ad-Ctbp2, Ad-Ctbp2+1 μmol/L KA, Ad-Ctbp2+5 μmol/L KA, Ad-Ctbp2+10 μmol/L KA cardiomyocytes (n = 5). Data were expressed as mean ± SD. *P < 0.05, ***P < 0.001, One-way ANOVA. **C** CCK8 analysis of control, Ad-NC, Ad-Ctbp2, Ad-Ctbp2+1:1 (Pyruvate:Lactate), Ad-Ctbp2+1:10 (Pyruvate:Lactate), Ad-Ctbp2+1:100 (Pyruvate:Lactate) cardiomyocytes (n = 5). Data were expressed as mean ± SD. *P < 0.05, ***P < 0.001, One-way ANOVA. **D** CCK8 analysis of control, Ad-NC, Ad-Ctbp2, Ad-Ctbp2+100 μmol/L Sodium Palmitate, Ad-Ctbp2+300 μmol/L Sodium Palmitate, Ad-Ctbp2+500 μmol/L Sodium Palmitate cardiomyocytes (n = 4). Data were expressed as mean ± SD. *P < 0.05, ***P < 0.001, One-way ANOVA. **E** EDU and cTnT immunofluorescence staining of control, Ad-NC, Ad-Ctbp2, Ad-Ctbp2+KA, Ad-Ctbp2+Lactate, Ad-Ctbp2+Sodium palmitate cardiomyocytes. Scale bar = 40 µm. **F** Statistical analysis of the number of EDU-positive cardiomyocytes (n ≥ 4). Data were expressed as mean ± SD. *P < 0.05, **P < 0.01, One-way ANOVA. **G** EDU and cTnT immunofluorescence staining of Ad-Ctbp2, Ad-Ctbp2+KA, Ad-Ctbp2+Lactate, Ad-Ctbp2+Sodium palmitate cardiomyocytes. Scale bar = 40 µm. **H** Statistical analysis of the percentage of RFP^+^EDU^+^ cardiomyocytes and RFP^+^EDU^−^ cardiomyocytes to RFP^+^cardiomyocytes

To validate our hypothesis, we overexpressed Ctbp2 in primary neonatal mice cardiomyocytes and subsequently added varying concentrations of koningic acid (KA), different ratios of lactate, and varying concentrations of sodium palmitate to the cell culture medium. This was done to modulate the levels of NADH/NAD+ and fatty acyl-CoAs in the cells and assess changes in the proliferation ability of cardiomyocytes (Fig. 6A). The CCK8 assay results showed that, compared to the control, overexpression of Ctbp2 promoted cardiomyocyte proliferation. Upon the addition of KA, cardiomyocyte proliferation was inhibited as the intracellular NADH decreased, with this inhibition gradually increasing with higher concentrations of KA (Fig. 6B). Conversely, the addition of the lactate solution led to an increase in NADH, which further promoted cardiomyocyte proliferation. This proliferative effect was enhanced with higher concentrations of the lactate solution (Fig. 6C). Following the addition of sodium palmitate, an increase in intracellular fatty acyl-CoAs inhibited cardiomyocyte proliferation, with this inhibition also increasing with higher concentrations of sodium palmitate (Fig. 6D). To visualize the regulatory effects of NADH/NAD+ and fatty acyl-CoAs on Ctbp2 in cardiomyocytes, we used EDU and cTnT immunofluorescence staining to further examine cell proliferation. The results indicated that, compared to Ad-NC cardiomyocytes, the number of EDU positive cardiomyocytes increased in Ad-Ctbp2 cardiomyocytes. In comparison to Ad-Ctbp2 cardiomyocytes, the number of EDU positive cardiomyocytes decreased in Ad-Ctbp2+KA and Ad-Ctbp2+Sodium palmitate cardiomyocytes, whereas the number of EDU positive cardiomyocytes further increased in Ad-Ctbp2+Lactate cardiomyocytes (Fig. 6E, F). In order to further study, we combined the Ad-Ctbp2-RFP expression in cardiomyocytes and found that in cardiomyocytes overexpressing Ctbp2 (RFP positive cardiomyocytes), approximately 52% were EDU positive, and about 48% were EDU negative. After adding KA, approximately 26% of RFP positive cardiomyocytes were EDU positive, and about 74% were EDU negative. Following the addition of lactate, approximately 61% of RFP positive cardiomyocytes were EDU positive, and about 39% were EDU negative. After the addition of sodium palmitate, approximately 21% of RFP positive cardiomyocytes were EDU positive, and about 79% were EDU negative (Fig. 6G, H).

These results indicate that the regulation of cardiomyocyte proliferation by Ctbp2 is influenced by the metabolites NADH/NAD+ and fatty acyl-CoAs. When Ctbp2 is overexpressed in primary neonatal mice cardiomyocytes, their proliferation capacity increases. However, when NADH levels decrease or fatty acyl-CoAs levels increase, the cardiomyocytes do not exhibit enhanced proliferation despite the overexpression of Ctbp2. Conversely, when NADH levels rise, the proliferation capacity of the cardiomyocytes is further enhanced, even on the basis of Ctbp2 overexpression, confirming our hypothesis.

### Metabolites NADH/NAD+ and acyl-CoAs affects the regulation of Ctbp2 on the cardiomyocytes proliferation may be achieved through the Ctbp2-FoxO1-p21/p27 pathways

**Fig. 7:**
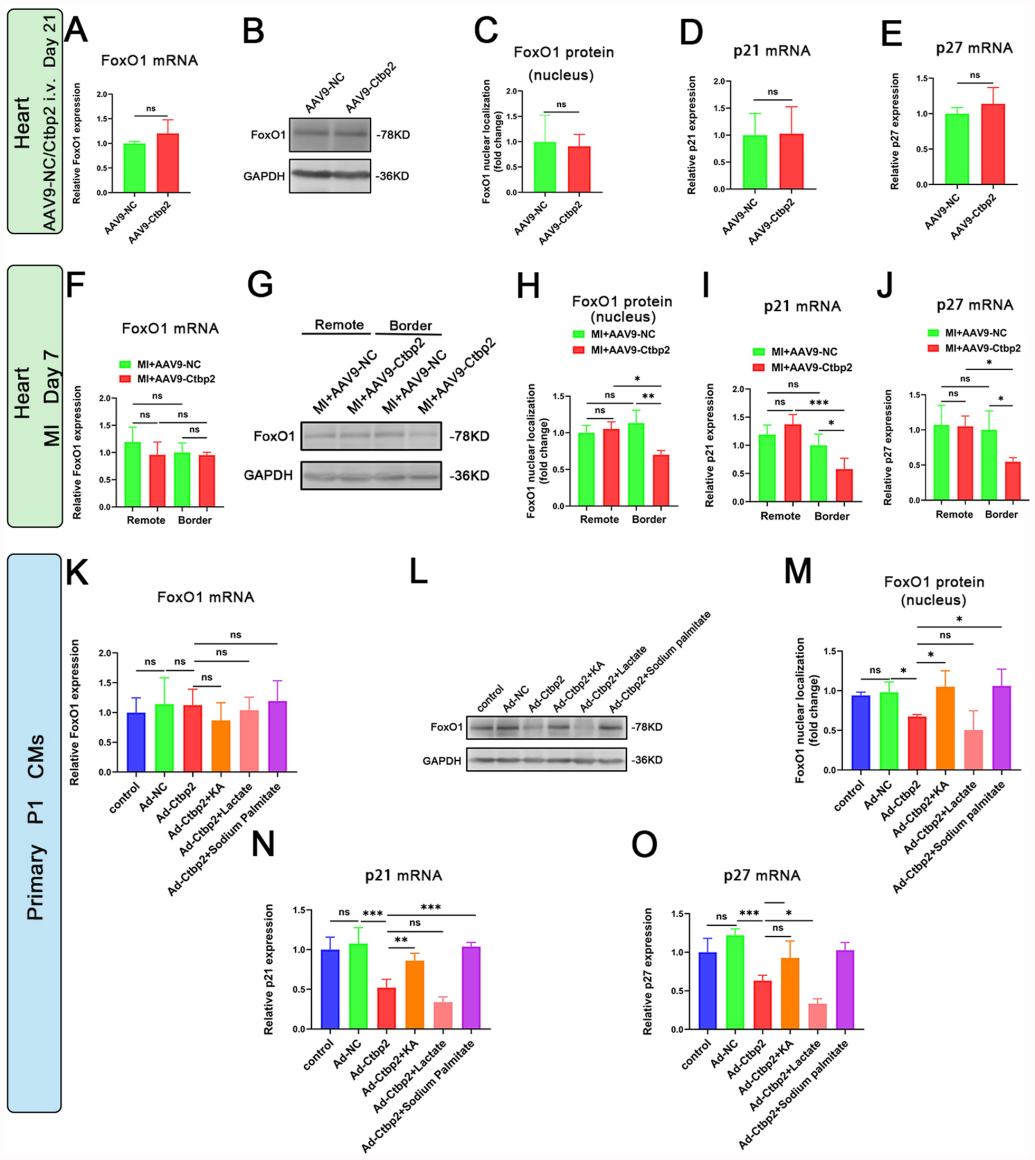
Metabolites NADH/NAD+ and fatty acyl-CoAs affects the regulation of Ctbp2 on the cardiomyocytes proliferation may be achieved through the Ctbp2-FoxO1-p21/p27 pathways. **A** qPCR analysis of FoxO1 in hearts from AAV9-NC and AAV9-Ctbp2 mice (n = 3). The data is represented as mean ± SD. t test. **B**, **C** Western-blot analysis of FoxO1 in heart tissue nucleus of AAV9-NC mice and AAV9-Ctbp2 mice (n = 3). The data is represented as mean ± SD. t test. **D**, **E** qPCR analysis of p21 and p27 in hearts from AAV9-NC and AAV9-Ctbp2 mice (n = 4). The data is represented as mean ± SD. t test. **F** qPCR analysis of FoxO1 in hearts from MI+AAV9-NC and MI+AAV9-Ctbp2 mice (n ≥ 3). Data were expressed as mean ± SD. One-way ANOVA. **G**, **H** Western-blot analysis of FoxO1 in heart tissue nucleus of MI+AAV9-NC mice and MI+AAV9-Ctbp2 mice (n ≥ 3). The data is represented as mean ± SD. *P < 0.05, **P < 0.01, One-way ANOVA. **I**, **J** qPCR analysis p21 and p27 in hearts from MI+AAV9-NC and MI+AAV9-Ctbp2 mice (n ≥ 3). Data were expressed as mean ± SD. *P < 0.05, ***P < 0.001, One-way ANOVA. **K** qPCR analysis of FoxO1 in control, Ad-NC, Ad-Ctbp2, Ad-Ctbp2+KA, Ad-Ctbp2+Lactate and Ad-Ctbp2+Sodium Palmitate cardiomyocytes (n = 4). Data were expressed as mean ± SD. One-way ANOVA. **L**, **M** Western-blot analysis of FoxO1 in nucleus of control, Ad-NC, Ad-Ctbp2, Ad-Ctbp2+KA, Ad-Ctbp2+Lactate and Ad-Ctbp2+Sodium Palmitate cardiomyocytes (n ≥ 3). Data were expressed as mean ± SD. *P < 0.05, One-way ANOVA. **N**, **O** qPCR analysis of p21 and p27 in control, Ad-NC, Ad-Ctbp2, Ad-Ctbp2+KA, Ad-Ctbp2+Lactate and Ad-Ctbp2+Sodium Palmitate cardiomyocytes (n ≥ 3). Data were expressed as mean ± SD. *P < 0.05, **P < 0.01, ***P < 0.001, One-way ANOVA

These results demonstrate that the metabolites NADH/NAD+ and fatty acyl-CoAs influence the regulation of cardiomyocyte proliferation by Ctbp2. To further investigate the underlying mechanisms, we examined the expression of the transcription factor FoxO1 and its target genes, the cell cycle negative regulators p21 and p27. First, in vivo experiments, we found that under normal conditions, there were no significant differences in the mRNA levels of FoxO1, p21, and p27, as well as the expression levels of the transcriptionally active FoxO1 protein in the nuclei of heart tissues between AAV9-NC and AAV9-Ctbp2 mice (Fig. 7A–E). In the context of heart injury, the mRNA levels of FoxO1, p21, and p27, along with the nuclear expression of FoxO1 protein, showed no significant differences in the remote area of heart tissue between MI+AAV9-NC and MI+AAV9-Ctbp2 mice. However, in the border area of heart tissue, compared to MI+AAV9-NC mice, MI+AAV9-Ctbp2 mice exhibited no change in FoxO1 mRNA levels, but a decrease in the nuclear expression of FoxO1 protein. Additionally, the mRNA levels of p21 and p27 were also reduced in the heart tissue (Fig. 7F–J). In vitro experiments, we observed that compared to Ad-NC cardiomyocytes, Ad-Ctbp2 cardiomyocytes showed no significant change in FoxO1 mRNA levels, but a decrease in the nuclear expression of FoxO1 protein. The mRNA levels of p21 and p27 were also reduced in these cells. Furthermore, compared to Ad-Ctbp2 cardiomyocytes, the addition of KA and sodium palmitate led to no significant change in FoxO1 mRNA levels, but an increase in the nuclear expression of FoxO1 protein, as well as an increase in the mRNA levels of p21 and p27. Conversely, the addition of lactate resulted in no significant change in FoxO1 mRNA levels, but a decrease in the nuclear expression of FoxO1 protein, and a reduction in the mRNA levels of p21 and p27 (Fig. 7K–O).

Combining the in vivo and in vitro results, the expression of FoxO1, p21, and p27 is correlated with the regulation of cardiomyocyte proliferation by Ctbp2. Specifically, when intracellular NADH levels increase, the transcriptional repressor Ctbp2 is activated in the nucleus, leading to a decrease in the expression of transcriptionally active FoxO1 protein. This suppresses the transcription of its target genes, the cell cycle negative regulators p21 and p27, thereby promoting cell proliferation. Conversely, when intracellular NADH levels decrease or fatty acyl-CoAs levels increase, Ctbp2 becomes inactivated in the nucleus, resulting in an increase in the expression of transcriptionally active FoxO1 protein. This enhances the transcription of its target genes, p21 and p27, thereby inhibiting cell proliferation. In summary, the metabolites NADH/NAD+ and acyl-CoAs affects the regulation of Ctbp2 on the cardiomyocytes proliferation may be achieved through the Ctbp2-FoxO1-p21/p27 pathways.

## Discussion

Although the mammalian heart has long been considered a terminally differentiated organ, numerous reports over the past few years have confirmed the generation of new cardiomyocytes in the postnatal hearts of mice and humans (Ali et al. 2014; Mollova et al. 2013; Senyo et al. 2012). However, the underlying mechanisms of postnatal cardiomyocyte proliferation remain largely unclear. It has been reported that certain proteins and microRNAs, such as Neuregulin 1, agrin, miRNA-17–92, and miRNA-708, regulate postnatal cardiomyocyte proliferation (Bassat et al. 2017; Chen et al. 2013; Deng et al. 2017; D’Uva et al. 2015). In adulthood, reactivating the dormant regenerative response observed in neonatal hearts may offer a therapeutic approach for cardiac repair, but this requires a deeper understanding of the mechanisms involved. However, prolonged regulation of these pathways could lead to hypertrophic cardiomyopathy or heart failure, as persistent inactivation of the Hippo signaling pathway can cause extensive cardiomyocyte dedifferentiation, impairing cardiac function and the repair of injured hearts post-myocardial infarction (D’Uva et al. 2015; Ikeda et al. 2019). Therefore, it is crucial to explore methods for controlled, transient regulation of these pathways to replace lost cardiomyocytes due to disease, thereby improving myocardial function after injury. This is the basis for our study.

**Fig. 8:**
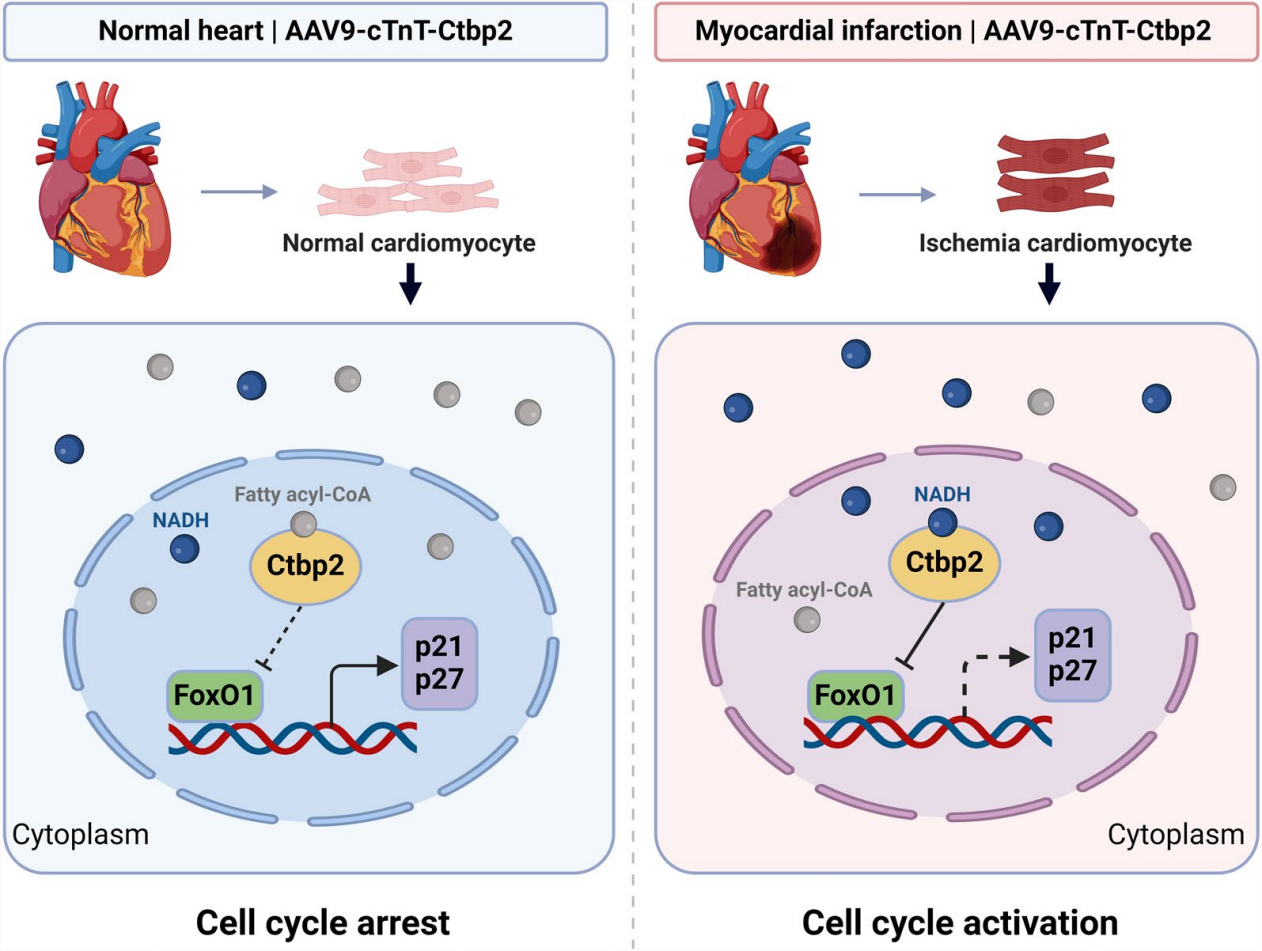
Schematic illustration on the signaling pathway of the transcriptional repressor Ctbp2 as a metabolite sensor regulating cardiomyocyte proliferation and heart regeneration

Ctbp2 is an evolutionarily conserved transcriptional repressor that regulates fundamental processes such as cell proliferation and apoptosis (Guan et al. 2013; Ju et al. 2021; Zhang et al. 2015). However, its potential role in regulating cardiomyocyte proliferation and heart regeneration remains unclear. In this study, we found that the expression of Ctbp2 in cardiomyocytes is downregulated in adulthood. Silencing Ctbp2 in P1 cardiomyocytes reduced their proliferation capacity, while overexpression of Ctbp2 enhanced cardiomyocyte proliferation. Additionally, AAV9-mediated overexpression of Ctbp2 had no effect on the hearts of normal adult mice, but promoted cardiomyocyte proliferation in the context of heart injury. Mechanistically, as a metabolite sensor, the transcriptional repressor Ctbp2 regulates cardiomyocyte proliferation is influenced by the metabolites NADH/NAD+ and fatty acyl-CoAs. When cardiomyocytes are ischemic or hypoxic, intracellular accumulation of NADH activates Ctbp2, inhibiting the transcriptional activity of the transcription factor FoxO1. This suppression leads to the downregulation of its target genes, the cell cycle negative regulators p21 and p27, thereby allowing the cardiomyocytes to re-enter the cell cycle. However, in normal adult cardiomyocytes, which primarily rely on fatty acid oxidation for energy, the intracellular accumulation of fatty acyl-CoAs inactivates Ctbp2. As a result, Ctbp2 is unable to suppress FoxO1 mediated cell cycle arrest (Fig. 8). Previous studies have explored how Ctbp2 perceives changes in intracellular metabolites. The interaction between Ctbp and transcription factors largely depends on the polymerization of Ctbp. When bound to NADH/NAD+, Ctbp forms a dimer or multimer configuration, which promotes the formation of transcriptional complexes (Bellesis et al. 2018; Bhambhani et al. 2011; 143. Saito et al. 2023). Structurally, the CoA portion of fatty acyl-CoA shares the same adenosine structure as NADH/NAD+, competing with NADH/NAD+ for the Rossmann fold pocket of Ctbp2, while the acyl chain portion is located at the interface of Ctbp2 dimer, physically preventing Ctbp2 dimerization (Sekiya et al. 2021).

FoxO1 inhibits the cell cycle through various mechanisms, including the activation of the cell cycle negative regulators p21 and p27 (Ho et al. 2008). Post-translational modifications can regulate the subcellular localization and transcriptional activity of FoxO (Maiese et al. 2009). Some major post-translational modifications include phosphorylation, acetylation, ubiquitination, arginine methylation, and O-glycosylation, which can either enhance or reduce the transcriptional activity of FoxO1 (Obsil and Obsilova 2008; Tzivion et al. 2011). In this study, we found that activated Ctbp2 can inhibit the transcriptional activity of FoxO1. The mRNA levels of FoxO1 remained unchanged in experiments, indicating that Ctbp2 does not affect the transcription of the FoxO1 gene itself. Furthermore, the change in the expression levels of the transcriptionally active FoxO1 protein in the nucleus may be related to the mechanism of Ctbp2 as a transcriptional repressor. Specifically, Ctbp2, as a transcriptional repressor lacking DNA-binding ability, binds to the transcription factor FoxO1 to access genomic regions. There, they recruit chromatin-modifying enzymes. These enzymes can alter chromatin structure through histone modifications, thereby suppressing the transcription of the target genes p21 and p27. Additionally, chromatin-modifying enzymes may also perform non-histone modifications on FoxO1, affecting its nuclear localization and reducing its protein expression levels in the nucleus, thereby inhibiting the expression of its target genes p21 and p27. However, whether Ctbp2 recruits chromatin-modifying enzymes to induce non-histone modifications in FoxO1 or what specific non-histone modifications occur in FoxO1 has not been further investigated in this study. Therefore, a clearer conclusion has not been obtained yet, and further experimental exploration is needed in the future.

In addition to activating the expression of the cell cycle negative regulators p21 and p27 to inhibit cardiomyocyte proliferation, FoxO1 also plays a key role in regulating cardiac glucose and fatty acid metabolism (Chistiakov et al. 2017). Studies have shown that FoxO1 promotes fatty acid oxidation in cardiac tissue while inhibiting glycolysis (Lei et al. 2013; Piao et al. 2012; Puthanveetil et al. 2011). Glycolysis is crucial for supporting rapid cell growth and proliferation in early stages, while oxidative metabolism provides sustained energy production and maintains the integrity and balance of mature cell types. Therefore, combining our research with previous reports, in conditions of myocardial hypoxia leading to NADH accumulation, NADH binds to Ctbp2 and activates it. Activated Ctbp2 then binds to FoxO1, inhibiting FoxO1 mediated metabolic pathways. This suppression inhibits fatty acid oxidation and promotes glycolysis. The enhanced glycolysis pathway promotes cardiomyocyte proliferation, and the glycolysis pathway generates more NADH, which can act on Ctbp2, activating it. This process forms a positive feedback regulation mechanism that continuously amplifies the role of Ctbp2 in promoting cardiomyocyte proliferation. However, further experimental exploration is needed to determine whether such a positive feedback regulation mechanism exists in this study. Whether there are changes in metabolism in adult cardiomyocytes after overexpression of Ctbp2, what changes occur, and whether the role of Ctbp2 in promoting cardiomyocyte proliferation can be continuously amplified has not been further explored in this study. Therefore, we have not yet obtained a clearer conclusion, and further experimental exploration is needed in the future.

## Conclusion

In summary, this study has identified a method for the controlled and transient regulation of cardiomyocyte proliferation through the study of Ctbp2. Specifically, in adult cardiomyocytes under ischemic and hypoxia conditions, the accumulation of NADH activates Ctbp2, thereby initiating its role in promoting cardiomyocyte proliferation. However, under normal conditions, adult cardiomyocytes primarily rely on fatty acid oxidation for energy, an increase in intracellular fatty acyl-CoAs leads to the inactivation of Ctbp2, preventing its role in promoting cardiomyocyte proliferation. This controlled and transient method of regulating cardiomyocyte proliferation avoids excessive regulation of cardiomyocytes, reducing the occurrence of side effects such as hypertrophic cardiomyopathy or heart failure. It provides new targets and perspectives for addressing the issues of cardiomyocyte proliferation and heart regeneration.

## Supplementary Information

Additional file 1.

Additional file 2.

## Data Availability

Not applicable. No datasets were generated or analysed during the current study.
