# Supplementary material for: The transcriptional repressor Ctbp2 as a metabolite sensor regulating cardiomyocytes proliferation and heart regeneration
Source: Mol Med. 2025 Mar 26;31:119. doi: 10.1186/s10020-025-01168-8 (PMC11948641; doi:10.1186/s10020-025-01168-8)
Supplement: Supplementary file 1 — Additional file 1. [file 10020_2025_1168_MOESM1_ESM.docx]

**
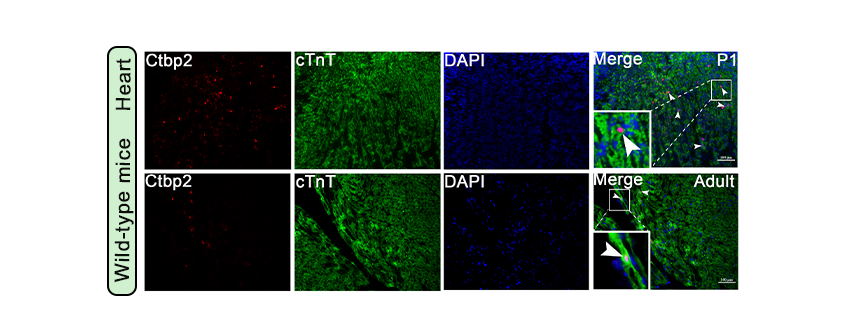
Fig. S1 Ctbp2 and WGA immunofluorescence staining of heart from P1 and Adult mice. Scale bar = 100µm.**


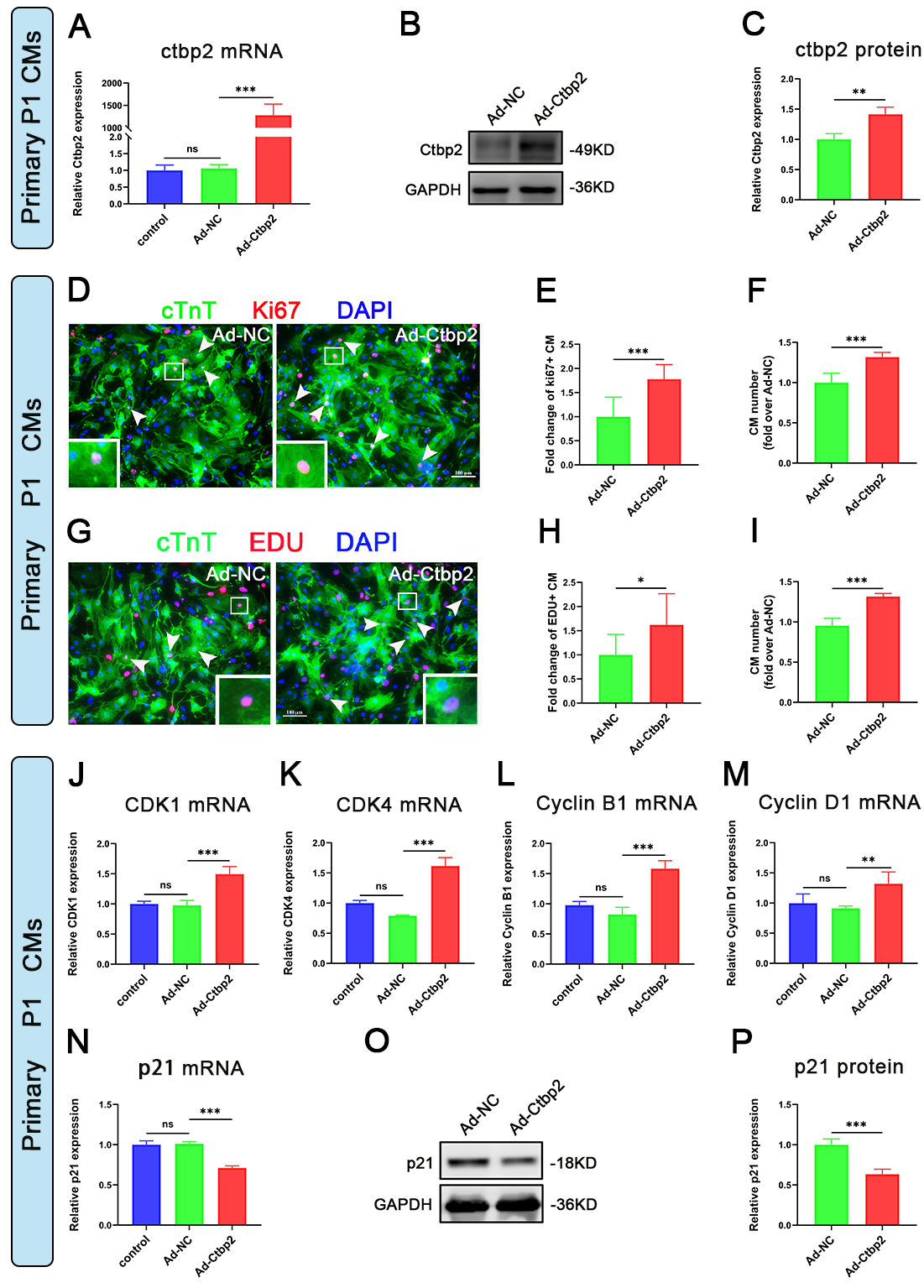


**Fig. S2 Overexpression of Ctbp2 promotes the proliferation of primary neonatal mice cardiomyocytes.** (A) qPCR analysis of Ctbp2 in cardiomyocytes from P1 mice after 72 hours of transfection with Ad-NC and Ad-Ctbp2. Data were expressed as mean ± SD (n ≥ 5). ****P* < 0.001, One-way ANOVA. (B, C) Western-blot analysis of Ctbp2 in cardiomyocytes from P1 mice after 72 hours of transfection with Ad-NC and Ad-Ctbp2 (n = 4). Data were expressed as mean ± SD. ***P* < 0.01, t test. (D) Ki67 and cTnT immunofluorescence staining of cardiomyocytes from P1 mice after 72 hours of transfection with Ad-NC and Ad-Ctbp2. Scale bar = 100µm. (E, F) Statistical analysis of the number of Ki67-positive cardiomyocytes and the total number of cardiomyocytes (n ≥ 9). Data were expressed as mean ± SD. ****P* < 0.001, t test. (G) EDU and cTnT immunofluorescence staining of cardiomyocytes from P1 mice after 72 hours of transfection with Ad-NC and Ad-Ctbp2. Scale bar = 100µm. (H, I) Statistical analysis of the number of EDU-positive cardiomyocytes and the total number of cardiomyocytes (n ≥ 5). Data were expressed as mean ± SD. **P* < 0.05, ****P* < 0.001, t test. (J-M) qPCR analysis of cell cycle activators (CDK1, CDK4, Cyclin B1 and Cyclin D1 ) in cardiomyocytes from P1 mice after 72 hours of transfection with Ad-NC and Ad-Ctbp2 (n ≥ 3). Data were expressed as mean ± SD. ***P* < 0.01, ****P* < 0.001, One-way ANOVA. (N) qPCR analysis of negative cell cycle regulators (p21) in cardiomyocytes from P1 mice after 72 hours of transfection with Ad-NC and Ad-Ctbp2 (n = 3). Data were expressed as mean ± SD. ****P* < 0.001, One-way ANOVA. (O, P) Western-blot analysis of negative cell cycle regulators (p21) in cardiomyocytes from P1 mice after 72 hours of transfection with Ad-NC and Ad-Ctbp2 (n ≥ 3). Data were expressed as mean ± SD. ****P* < 0.001, t test.


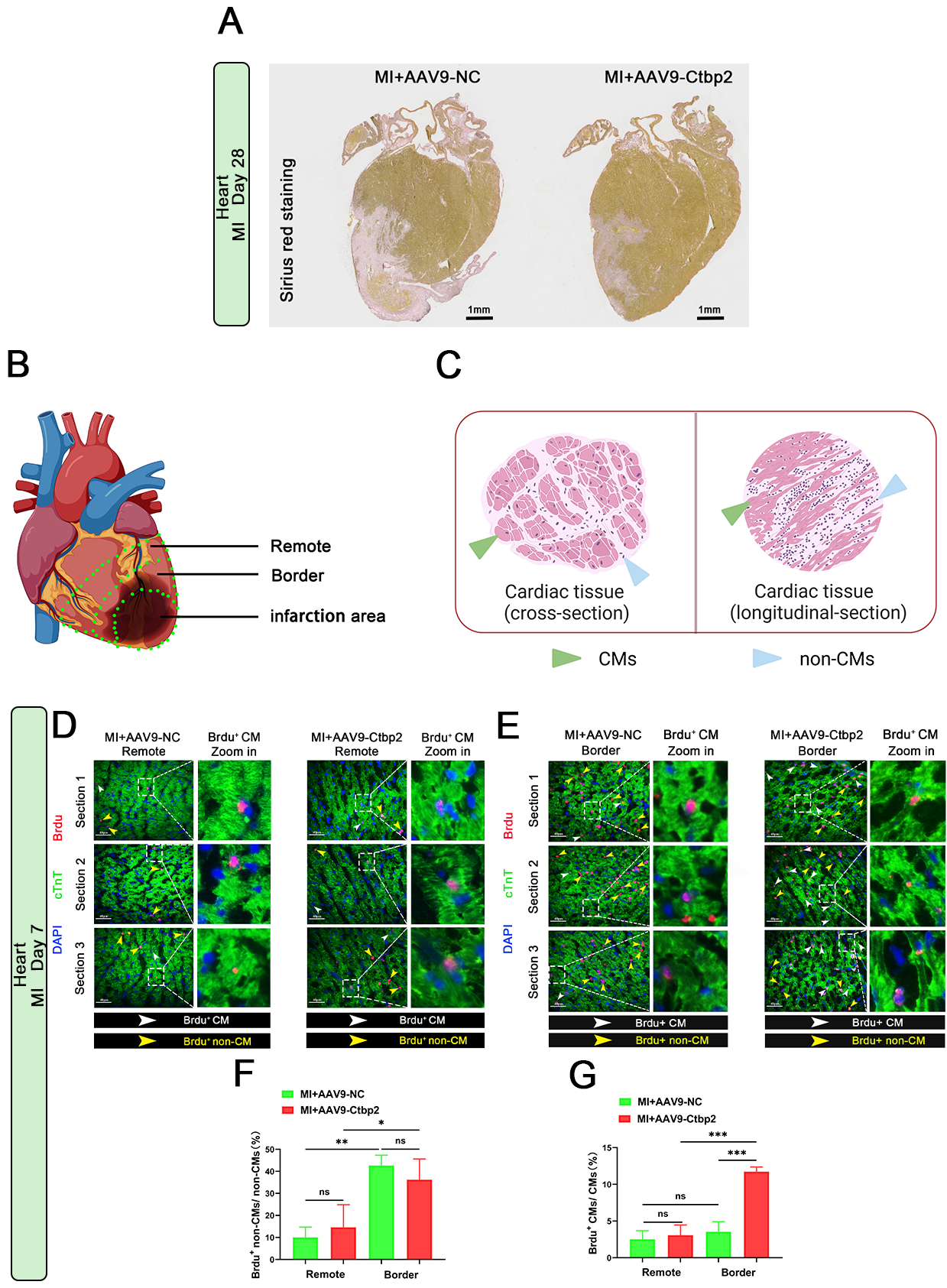


**Fig. S3 Overexpression of Ctbp2 promotes cardiomyocyte proliferation on adult mice with heart injury.** (A) Sirius red staining of heart from MI+AAV9-NC mice and MI+AAV9-Ctbp2 mice. Scale bar = 1mm. (B) Infarcted heart zoning (infarction area, Border, Remote) pattern map. (C) Schematic diagram for distinguishing cardiomyocytes and non-cardiomyocytes in cardiac tissue. (D, E) Brdu and cTnT immunofluorescence staining of heart from MI+AAV9-NC mice and MI+AAV9-Ctbp2 mice. Scale bar = 40µm. (F, G) Statistical analysis of the proportion of Brdu-positive non-cardiomyocytes and Brdu-positive cardiomyocytes (n = 3). Data were expressed as mean ± SD. **P* < 0.05, ***P* < 0.01, ****P* < 0.001, One-way ANOVA.
