# Supplementary figures and images for: The transcriptional repressor Ctbp2 as a metabolite sensor regulating cardiomyocytes proliferation and heart regeneration
Source: Mol Med. 2025 Mar 26;31:119. doi: 10.1186/s10020-025-01168-8 (PMC11948641; doi:10.1186/s10020-025-01168-8)

**Fig. 1D**

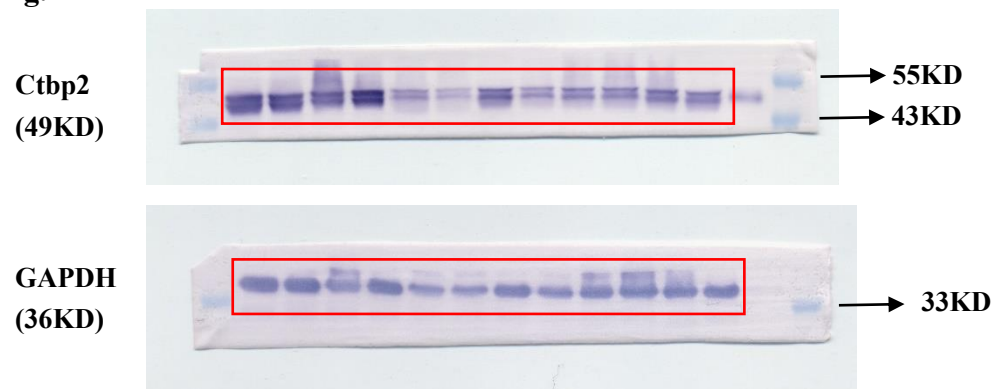

**Fig. 2B**

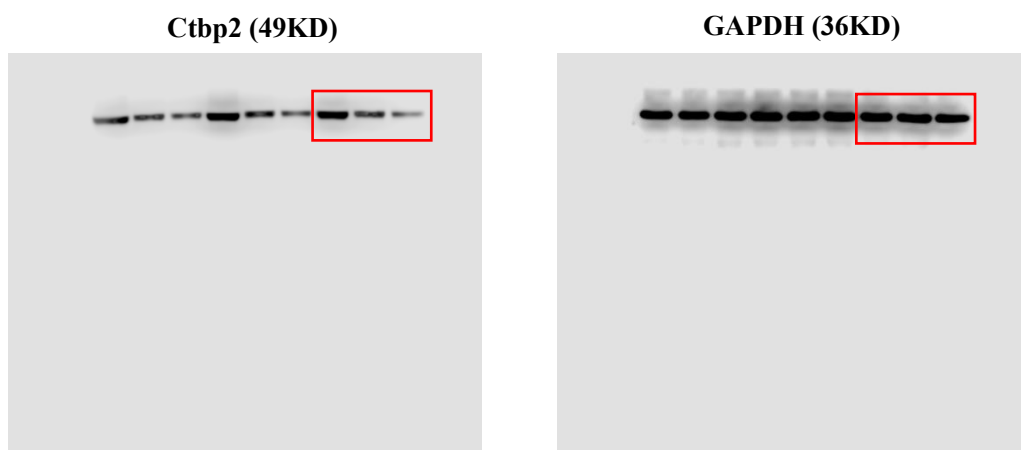

**Fig. 2O**

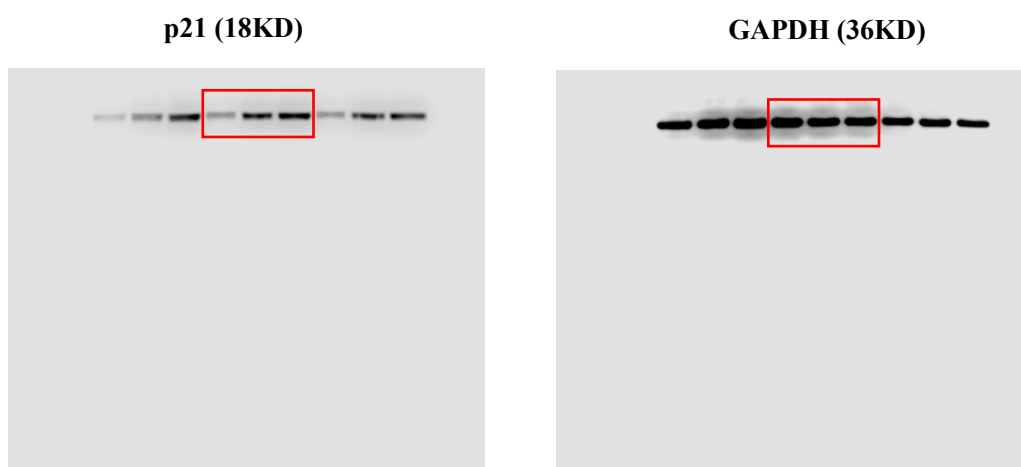

**Fig. 3C**

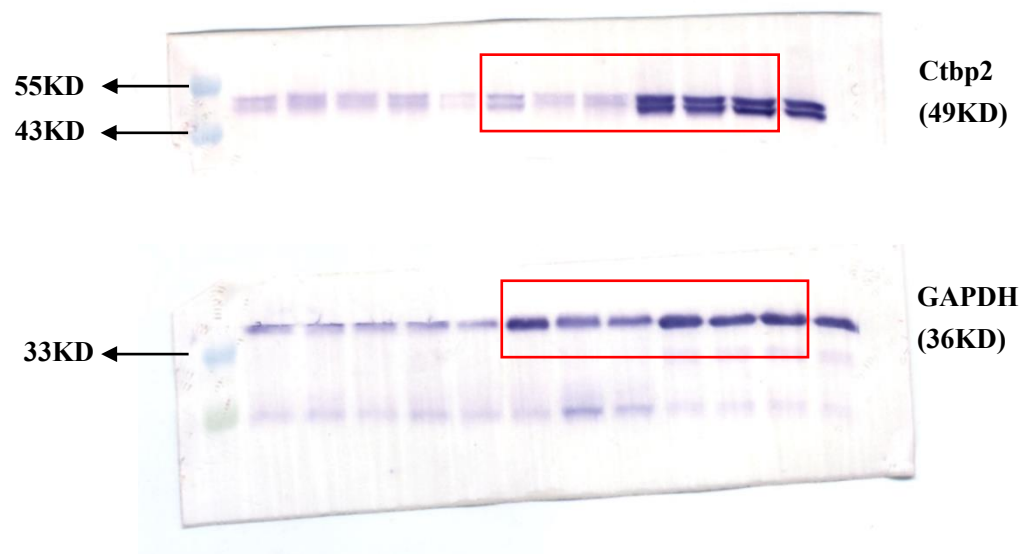

**Fig. 7B**

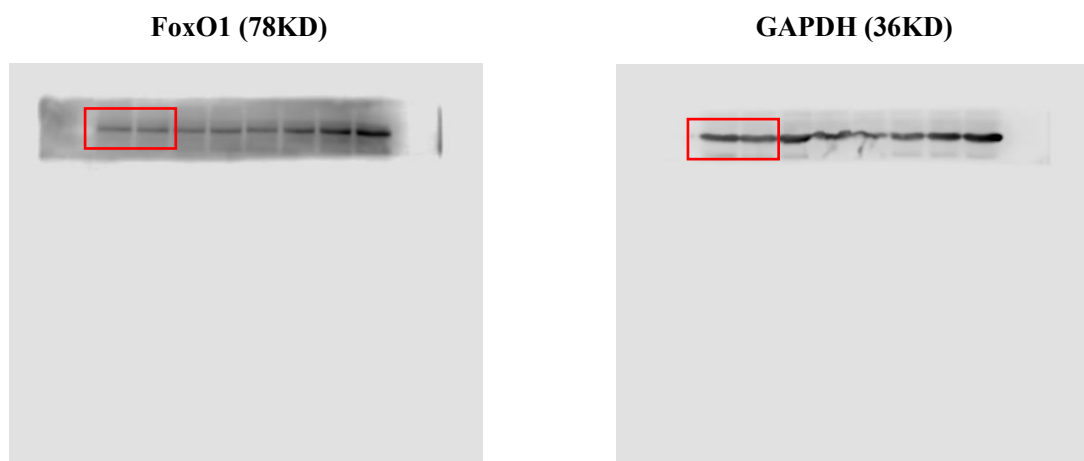

**Fig. 7G**

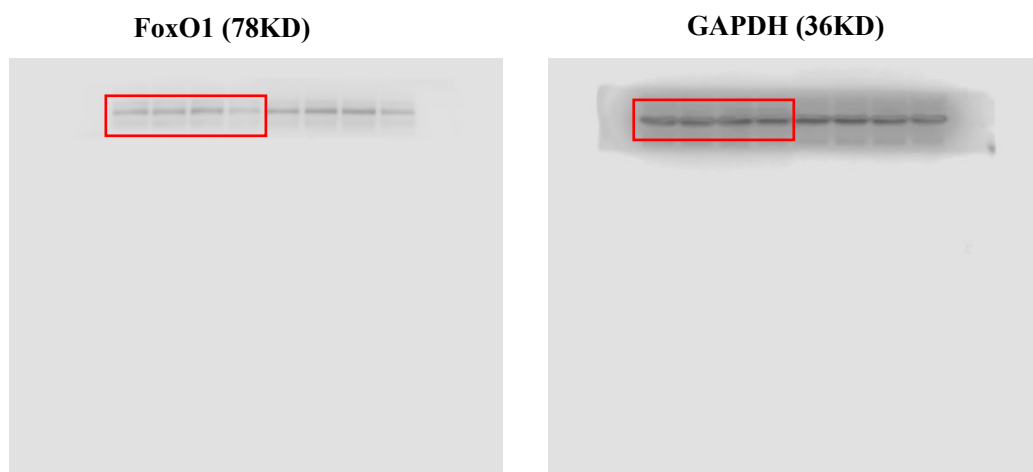

**Fig. 7L**

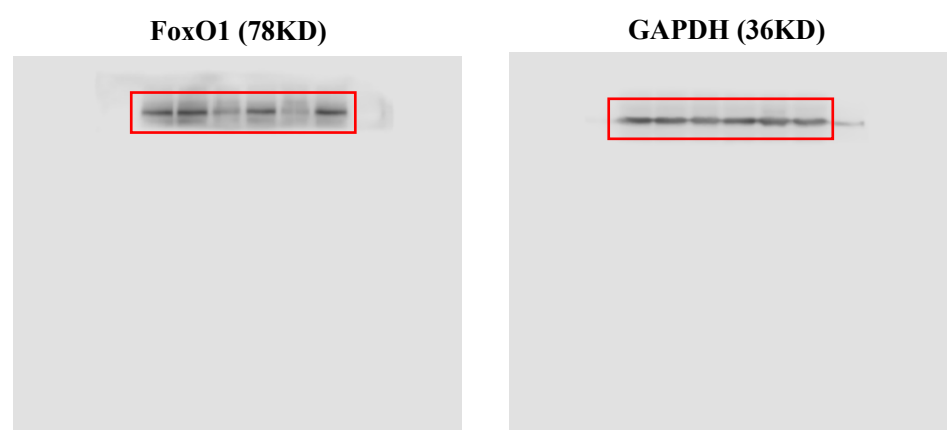

**Fig. S2B**

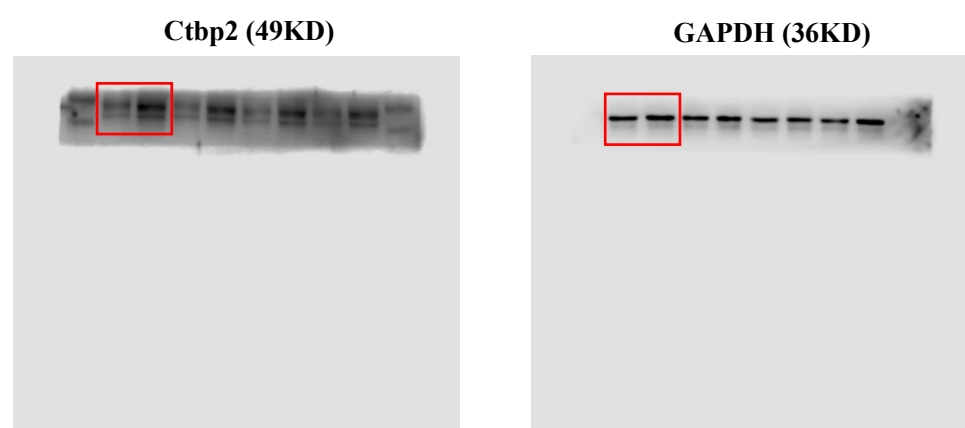

**Fig. S2O**

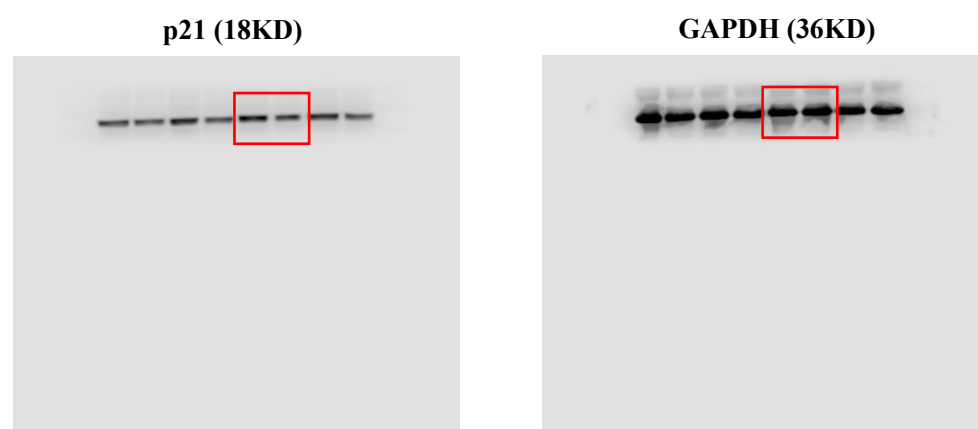

Supplement: Supplementary file 2 — Additional file 2. [file 10020_2025_1168_MOESM2_ESM.pdf]
